# New characterization and safety evaluation of human limbal stem cells used in clinical application: fidelity of mitotic process and mitotic spindle morphologies

**DOI:** 10.1186/s13287-023-03586-z

**Published:** 2023-12-13

**Authors:** Marija Zekušić, Marina Bujić Mihica, Marija Skoko, Kruno Vukušić, Patrik Risteski, Jelena Martinčić, Iva M. Tolić, Krešo Bendelja, Snježana Ramić, Tamara Dolenec, Ivana Vrgoč Zimić, Dominik Puljić, Ivanka Petric Vicković, Renata Iveković, Ivanka Batarilo, Uršula Prosenc Zmrzljak, Alan Hoffmeister, Tiha Vučemilo

**Affiliations:** 1 Department of Transfusion and Regenerative Medicine, Sestre milosrdnice University Hospital Center, Zagreb, Croatia; 2 Division of Molecular Biology, Ruđer Bošković Institute, Zagreb, Croatia; 3 Center for Research and Knowledge Transfer in Biotechnology, Laboratory of Immunology, University of Zagreb, Zagreb, Croatia; 4 Department of Oncological Pathology and Clinical Cytology ‘Ljudevit Jurak’, University Hospital Center Sestre Milosrdnice, Zagreb, Croatia; 5 Clinical Department of Ophthalmology, Sestre milosrdnice University Hospital Center, Zagreb, Croatia; 6 Department of Microbiology, Croatian Institute of Transfusion Medicine, Zagreb, Croatia; 7 Molecular Biology Department, BIA Separations CRO, Labena d.O.O, Ljubljana, Slovenia; 8 Labena d.o.o, Zagreb, Croatia; 9 Charles River Laboratories, Canterbury, UK

**Keywords:** Limbal stem cell, Limbal stem cell deficiency, Limbal graft, Advanced therapy medicinal products, p63

## Abstract

**Background:**

Limbal stem cells (LSCs) are crucial for the regeneration of the corneal epithelium in patients with limbal stem cell deficiency (LSCD). Thus, LSCs during cultivation in vitro should be in highly homogeneous amounts, while potency and expression of stemness without tumorigenesis would be desirable. Therefore, further characterization and safety evaluation of engineered limbal grafts is required to provide safe and high-quality therapeutic applications.

**Methods:**

After in vitro expansion, LSCs undergo laboratory characterization in a single-cell suspension, cell culture, and in limbal grafts before transplantation. Using a clinically applicable protocol, the data collected on LSCs at passage 1 were summarized, including: identity (cell size, morphology); potency (yield, viability, population doubling time, colony-forming efficiency); expression of putative stem cell markers through flow cytometry, immunofluorescence, and immunohistochemistry. Then, mitotic chromosome stability and normal mitotic outcomes were explored by using live-cell imaging. Finally, impurities, bacterial endotoxins and sterility were determined.

**Results:**

Expression of the stemness marker p63 in single-cell suspension and in cell culture showed high values by different methods. Limbal grafts showed p63-positive cells (78.7 ± 9.4%), Ki67 proliferation (41.7 ± 15.9%), while CK3 was negative. Impurity with 3T3 feeder cells and endotoxins was minimized. We presented mitotic spindles with a length of 11.40 ± 0.54 m and a spindle width of 8.05 ± 0.55 m as new characterization in LSC culture. Additionally, live-cell imaging of LSCs (n = 873) was performed, and only a small fraction < 2.5% of aberrant interphase cells was observed; 2.12 ± 2.10% of mitotic spindles exhibited a multipolar phenotype during metaphase, and 3.84 ± 3.77% of anaphase cells had a DNA signal present within the spindle midzone, indicating a chromosome bridge or lagging chromosome phenotype.

**Conclusion:**

This manuscript provides, for the first time, detailed characterization of the parameters of fidelity of the mitotic process and mitotic spindle morphologies of LSCs used in a direct clinical application. Our data show that p63-positive CK3-negative LSCs grown in vitro for clinical purposes undergo mitotic processes with extremely high fidelity, suggesting high karyotype stability. This finding confirms LSCs as a high-quality and safe therapy for eye regeneration in humans.

## Background

Scientific progress in the fields of cell biology, molecular biotechnology and ocular regenerative medicine has contributed to the development of cell-based therapies for ocular surface rehabilitation over the last three decades. The cornea is the protective front part of the eye with transparent, avascular, stratified tissue, and these characteristics are essential for maintaining good vision. The limbus forms the border between the cornea and the conjunctiva and is the anatomical location of the stem cells responsible for maintaining a clear and transparent corneal epithelium [1]. Human limbal stem cells (LSCs) are located in the deep basal layer of the limbus and act as a reservoir for the corneal epithelium and as a barrier against the invasion of conjunctival epithelium over the corneal surface [2]. LSCs share some common features with other adult stem cells, such as small cell size [3], slow cycling [1], and expression of stem cell-associated markers such as p63, particularly the ΔNp63α isoform [4] and ATP-binding cassette subfamily G member 2 (ABCG2) [5], but lack markers for larger, terminally differentiated corneal cells such as cytokeratin 3 (CK3). Therefore, the dysfunction and/or insufficient quantity of LSCs irreversibly lead to pain, decreased vision, and photophobia [6].

Limbal stem cell deficiency (LSCD) is a rare, progressive blinding corneal disorder that can be caused by thermal or chemical injury, contact lens wear, multiple surgeries involving the limbus, infectious ocular disease, severe pterygium, etc. [7]. According to a previous review and meta-analysis of globally reported cases, chemical injury is by far the most common cause of LSCD, responsible for at least 66–75% of cases [8]. Additionally, LSCD can occur in autoimmune diseases such as Stevens-Johnson syndrome with developed infectious keratitis as a common complication [9]. All the above-mentioned conditions, caused by injury or autoimmune disease, reduce the LSC population, and in their absence, the conjunctival epithelium migrates across the cornea, causing connective spread that leads to complete loss of vision [10]. More than 10 million people worldwide are affected by LSCD [11].

Different approaches have been used in LSCD therapy with the general goal of regenerating the corneal epithelium. Autologous cultivated limbal epithelial transplantation (CLET) remains one of the most successful therapeutic approaches for the treatment of patients with LSCD to date. By definition, CLET fits the advanced therapy medicinal products (ATMPs) classification defined as a tissue-engineering product according to European regulation no. 1394/2007 with the capability to repair, regenerate or replace human tissue [12].

The techniques commonly used to test products that are applied in regenerative medicine are flow cytometry, immunocytochemistry, immunohistochemistry, and Western blotting. The use of these techniques represents the gold standard in regenerative medicine clinical practice that has led to massive advancement in the field in the past decade [13]. However, we still lack a detailed examination of the processes that occur in LSCs in the environment in which they are grown, especially during a short time period when stem cells divide and build the substrate for autologous cultivated limbal epithelium that will be used for transplantation [14]. Confocal fluorescence microscopy, a high-resolution single-cell imaging technique, which is still not widely used in the ATMP field [15], would produce quantitative information on cellular processes as they occur during LSC culture. As accurate chromosome segregation during cell division and thus mitotic fidelity of cells used in CLET depend on the assembly of a bipolar mitotic spindle, a cellular micromachine made of microtubules that connects sister chromatids to each spindle pole [16], it is of particular interest to image this structure at high resolution. The development of superior microscopy approaches in recent years has enabled unprecedented examination of the details of the mitotic process and its dynamics during cell division [17]. Thus, acquiring data on the fidelity of mitotic mechanisms in highly relevant and clinically used samples of LSCs could bridge the work of cell biologists and clinicians by combining relevant clinical approaches used on patients with recent advances in cell biology techniques.

For more than two centuries, it has been known that every cell must divide to produce its offspring and pass the genetic material to the next generation of cells [18]. During the anaphase of cell division, the mitotic spindle drives physical separation of a complete set of chromosomes into two equal parts destined for the two daughter cells [19]. Moreover, mitosis is essential not only for cell propagation, but its precision is vital for the normal life of the cell and thus for the normal homeostasis of the whole organism [20]. Although aneuploidy is often detrimental at the cellular level, it is a hallmark of human cancers since approximately 90% of tumors have gained or lost at least one chromosome, thus producing an imbalance from the normal diploid karyotype [21]. Furthermore, regarding broader cell therapy methods in the field of endothelial corneal diseases it was reported in cultured human corneal endothelial cells that the karyotype imbalance correlates both with the age of the donor and the extent of cell passaging of corneal cells [22]. Therefore, it is of utmost importance to score the number of cells with common mitotic defects, defective interphase nuclei, and micronuclei during LSC culture. In that way, one could check for negative effects of extensive cell passaging in culture on mitotic fidelity with the goal of ensuring greater security of limbal graft preparation procedures. Furthermore, data on mitotic spindles in general are scarce in stem cells, especially in LSCs, and therefore, it is of interest to quantify the basic parameters of the mitotic process and mitotic spindle morphologies of LSCs grown in culture during preparation of limbal grafts to establish the basic parameters of LSC biology that could be relevant for CLET usage in clinical practice.

With respect to CLET outcomes, the characterization and safety of LSCs was evaluated in this study in a single-cell suspension, in a two-dimensional (2D) cell culture, and in a three-dimensional (3D) limbal graft as a tissue engineered product. Such evaluation of LSC phenotypes, including identity, potency, stemness, proliferation rate and tumorigenesis, was performed as part of rigorous safety control. Furthermore, there is limited knowledge about the length and width of mitotic spindles during the cultivation of LSCs for therapeutic applications, the segregation of chromosomes, and the mitotic aberrations that were investigated in this study.

## Methods

### Study design

**Fig. 1:**
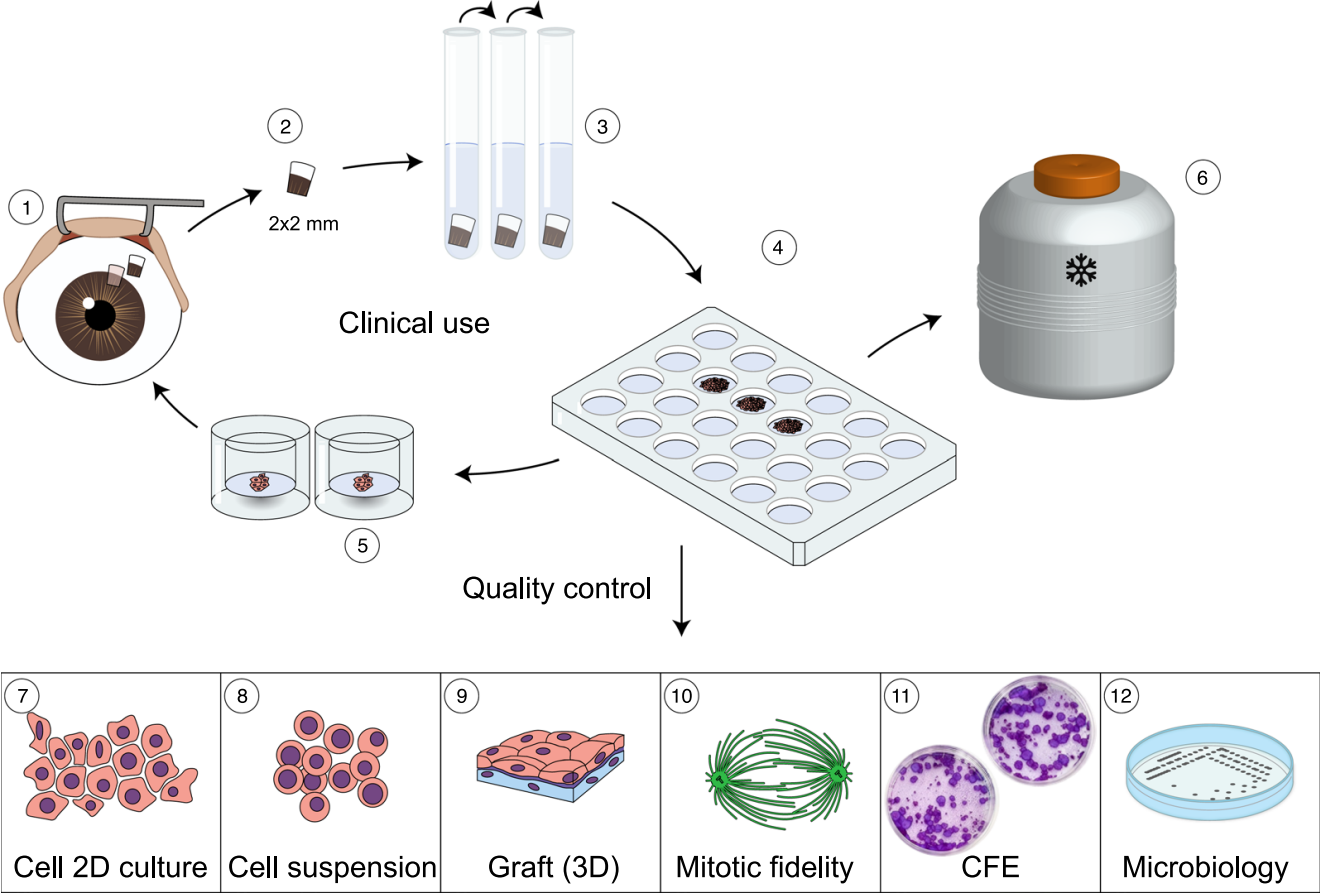
Schematic standard protocol of LSCs prepared for therapeutic applications. The protocol includes performing a limbus biopsy from the healthy eye (**1**, **2**), triple enzymatic digestion of the limbus (**3**), seeding of LSCs in fields of the cultivation vessel with 24 fields (**4**), and preparation of two limbal grafts for clinical application (**5**). A portion of the LSCs was stored in liquid nitrogen for potential recultivation (**6**), while the remaining cells were used for analysis as part of routine quality control that included expression of different markers in 2D culture, in the single-cell suspension and in the limbal graft as 3D culture (**7**, **8**, **9**), cytokinesis following 3D mitotic spindles and live-cell four-dimensional (4D) imaging (**10**), yield, viability, population doubling time, colony-forming efficiency (CFE) (**11**) and evaluation of microbiological sterility and the level of bacterial endotoxins (**12**)

The research study was approved by the Ethics Committee of Sestre milosrdnice University Hospital Center, Zagreb, Croatia (December 29th, 2014, EP-15333/14), and the research was performed in accordance with the Declaration of Helsinki. These procedures were performed with the approval of the competent authority, i.e., the Croatian Ministry of Health, as “hospital exemption” (date of approval August 20, 2018). The Tissue and Cell Bank complies with quality and safety standards for the donation, procurement, testing, processing, preservation, storage and distribution of human tissues and cells for clinical use. A schematic representation of the research study is illustrated in Fig. 1.

### Limbus processing procedures

Limbal biopsy (2 × 2 mm^2^) was performed on the healthy eyes of adult patients in the period from July 2019 to May 2022. The biopsy of the superior limbus region (corneoscleral tissues of limbal rings) was taken aseptically in the operating room with local anesthesia at the Department of Ophthalmology, University Hospital Center Sestre Milosrdnice, Zagreb, Croatia. Transport of the biopsy specimen from the operating room to the Tissue and Cell Bank was carried out using a 4 mL sterile tube (Techno Plastic Products AG, Trasadingen, Switzerland) with 2.5 mL growth medium (GM) (Dulbecco's Modified Eagle Medium (DMEM) and Ham’s F12 media (2:1 mixture) containing heat-inactivated fetal bovine serum (FBS) (10%) (Gibco, Thermo Fisher Scientific), with added growth supplements: 4 mM L-glutamine (Gibco, Thermo Fisher Scientific), 0.19 mM adenine (Sigma‒Aldrich, Saint Louis, USA), 5 µg/mL insulin (Sigma‒Aldrich, Saint Louis, USA), 0.1 nM cholera toxin (List Biological Laboratories, Campbell, California, USA), 1.36 ng/mL triiodothyronine (2 nM**)** (Sigma‒Aldrich, Saint Louis, USA), 0.4 μg/mL hydrocortisone (Sigma‒Aldrich, Saint Louis, USA), 5 μg/mL apotransferrin (Sigma‒Aldrich, Saint Louis, USA), 10 μg/mL human epidermal growth factor (EGF) (Sigma‒Aldrich, Saint Louis, USA) and 1% penicillin‒streptomycin-amphotericin B (100 lU/mL) (Gibco, Thermo Fisher Scientific).

### Preparation of the feeder layer

All procedures were performed under aseptic conditions in a microbiological safety cabinet (MSC) with a grade B background (ISO class 5 environment) in a Laboratory for Tissue Engineering. Briefly, the feeder layer was prepared from a 3T3 murine Swiss albino fibroblast cell line (ATCC® CCL-92TM, VA, USA) preferably for a minimum of two hours and a maximum of 24 h before limbal cell seeding. The 3T3 cells were used at passages 6 to 10 and cultivated in fibroblast medium (basal DMEM containing 10% FBS and 1% penicillin‒streptomycin-amphotericin B). The medium was filtered with a 0.22 µm filter (Techno Plastic Products AG, Trasadingen, Switzerland). When the 3T3 cells reached 80% confluence, as determined by phase contrast microscopy (Nikon GMHB, Eclipse Ti, Germany), they were treated with 0.5 mg/mL mitomycin C (Sigma‒Aldrich, Saint Louis, USA) for a minimum of two and a half and a maximum of three hours at 37 °C. The cells were washed three times with calcium and magnesium free Dulbecco’s phosphate buffer saline (DPBS) (Sigma‒Aldrich, Saint Louis, USA) and detached using 0.05% trypsin/0.02% EDTA (ethylenediaminetetraacetic acid) (Sigma‒Aldrich, Saint Louis, USA). Trypsin was neutralized with the addition of the same amount of GM and centrifuged at 200 × g for 4 min at 4 °C. The cells were resuspended in GM and seeded at a concentration of 2.4 × 10^4^ cells/cm^2^. They were unable to divide but were viable to secrete growth factors and cytokines, ensuring an optimal microenvironment for limbal cells to proliferate.

### Isolation of limbal stem cells

Upon delivery, limbus biopsies were decontaminated by immersing the tissue in a 5% solution of penicillin‒streptomycin-amphotericin B in DPBS four times for 10 min at room temperature. After decontamination, the tissue was immersed in a 15 mL polypropylene tube filled with 2 mL of 0.05% trypsin/0.02% EDTA (tube labeled 1′) and incubated in a water bath at 37 °C for 30 min. During incubation, the tube was vortexed occasionally with the orbital mixer to obtain single-cell suspensions. After 30 min of incubation, the tissue was transferred to a new 15 mL tube, and the incubation procedure was repeated two times (tube 2′ and tube 3′) with occasional vortexing. Suspensions of isolated limbal cells containing 2 mL of 0.05% trypsin/0.02% EDTA were neutralized by the addition of 4 mL of GM and centrifuged at 200 × g for five minutes at 4 °C. The same procedure had to be repeated with tubes 2 and 3. In brief, single-cell suspensions were obtained by a three-step enzymatic digestion and seeded on a prepared feeder layer in a 24-well plastic plate at a density of 1.53 × 10^4^ cells/cm^2^. After plating the limbal cells, the 24-well culture dish was labeled with the following information: tissue code, passage number, field where the cells were seeded, date of plating, and operator's initials. The suspension of limbal cells isolated from limbus biopsies was labeled as the primary passage (p0). The cell cultures were incubated at 37 °C and 5% CO_2_ in GM.

### Preparation of two limbal grafts as tissue-engineered products for clinical use

Seven to ten days after isolation, limbal cell cultures were 80% confluent, and they were ready for expansion in secondary cultures. Denudated human amniotic membranes (hAMs) that were used as LSC carriers were prepared in our tissue bank. Informed consent from women was obtained with proper serological and molecular screening. Generally, the placenta was obtained from healthy pregnant patients undergoing scheduled cesarean sections. After mechanical separation of the amnion from the chorion and decontamination with BASE 128 (AL.CHI.MI.A. S.r.l, Italy) and rinsing with BASE (AL.CHI.MI.A. S.r.l, Italy), the pieces of hAM were fixed on a nitrocellulose membrane (stromal side up) and cryopreserved in 90% BASE medium and 10% dimethyl sulfoxide (DMSO) (AL.CHI.MI.A. S.R.L., Ponte San Nicolo, Italy). Before use for the LSC culture, two 5 × 5 cm pieces of hAM were thawed, washed three times in saline and treated with a swab immersed in 0.5 M NaOH (0.12 mg/mL) (Sigma‒Aldrich, Saint Louis, USA) for 30 s to remove the amniotic epithelium. After that procedure, called denudation, the hAM was washed in DPBS, and the orientation of the membrane was tested with a cotton swab. Denuded hAMs with a de-epithelized surface on top were fixed in an interlock able sterile plastic ring CellCrown™6 (Merck KGaA, Darmstadt, Germany) with a 5.3 cm^2^ inner surface area and placed into a 6-well culture plate, and culture medium was added. Next, 3T3 cells (2.44 × 10^5^) were seeded on the denuded epithelial side of the hAM, and then, two to 24 h later, limbal cells (1.44 × 10^5^) were seeded on top of the 3T3 cells and prepared as a 3D limbal graft. Limbal graft cultivation was continued in GM in an incubator at 37 °C with 5% CO_2_. The GM was changed every three days. On the day of clinical application, the GM was replaced with 3–4 mL of medium L-15 (Leibovitz) (Sigma‒Aldrich, Saint Louis, USA). Two limbal grafts were packaged in appropriate primary and secondary packaging. The primary packaging was a 6-well culture dish wrapped in parafilm, and the secondary packaging was a sterile plastic zip bag. There was a label on the package that contained the patient's name and surname, tissue code, type of tissue, date and time of packaging with the additional label "for autologous use only". Transport of the limbal grafts from the tissue bank to the Department of Ophthalmology was carried out at temperatures from + 2 °C to + 8 °C in a validated shipping container.

### Quality control methods

#### Cell count and viability assay, colony-forming efficiency

Cell counts and viability were determined by Trypan blue staining using a Neubauer chamber and phase-contrast microscopy. To estimate the total number of cells, one portion of 0.4% Trypan blue dye and an equal portion of the original cell suspension were added, mixed and incubated for three minutes at room temperature. The ratio of cell suspension to Trypan blue was 1:1, and the dilution factor was 2. The cell count and viability analysis were performed visually under a light microscope. Viable cells were living cells with a circular shape, clear cytoplasm and an intact cell membrane (unstained), while dead cells had blue cytoplasm. Only viable cells were counted in four corner squares with 16 squares each in the Neubauer chamber. The calculation of the total number of cells per milliliter of cell suspension was performed as follows:

Total number of cells = count living cells × correction factor (10^4^) × dilution factor (2) × volume of the original cell suspension. Calculation of cell viability (%) was performed according to the formula:

$$Cell\, viability (\%)=\frac{\mathrm{total\, number\, of\, unstained\, cells }\,(\mathrm{viable}) }{\mathrm{total\, number\, of\, cells }\,(\mathrm{blue\, stained }+\mathrm{ unstained})}x100$$

Colony-forming efficiency (CFE) was examined by inoculation of 2 × 10^6^ 3T3 cells prepared as a feeder layer and 1500 LSCs seeded in parallel in two Petri dishes with an internal diameter of 87 mm, i.e., a growth surface 60 cm^2^ (Techno Plastic Products AG, Trasadingen, Switzerland). Cell growth was monitored daily on a microscope and analyzed on day 14. The remaining feeder layer was washed first with DPBS and then with 0.02% EDTA for 30 s (Sigma‒Aldrich, Saint Louis, USA). The cells were then fixed in 4% paraformaldehyde (PFA) (Santa Cruz Biotechnology, Inc., Heidelberg, Germany), stained with 0.05% crystal violet (Sigma‒Aldrich, Saint Louis, USA) for 30 min and washed with DPBS. CFE was calculated according to the formula:

$$CFE\, (\%)=\frac{\mathrm{the\, number\, of\, colonies}}{\mathrm{total\, number\, of\, seeded\, cells}}x100$$

The colonies were categorized into three types (holoclones, meroclones, paraclones) based on their morphology and size, as described by Barrandon Y and Green H.[23]. In summary, holoclones were identified as large, circular colonies with a smooth perimeter and an area of ≥ 10 mm^2^. Meroclones were slightly smaller, with a size area of 5–9 mm^2^ and a wrinkled perimeter. Paraclones were the smallest, with a diameter ≤ 4 mm^2^ and a highly irregular perimeter [24]. Colony size was measured in millimeters.

### Cumulative population doubling and doubling time

The calculation of cumulative population doubling (cPDs) and doubling time (DT) was performed when cells reached 80–90% confluency as follows:

$$cPDs = \log \frac{N}{N0}x3.33 \quad DT = \frac{CT}{{cPDs}}$$

where *N* is the number of live cells trypsinized in subconfluence, *N*_0_ is the initial number of live cells seeded, and CT is the total number of culture days [25].

### Immunocytochemical staining

To confirm the expression of the stem cell-associated marker p63, an additional immunocytochemical (ICC) staining assay was performed. To choose the best method to assess the quality of the cultured cells/limbal graft, during the analysis of the first sample, the cultured cells were stained with two clones for p63 (data not shown). The first clone covers ∆Np63α specific for LSC (ab735, Abcam, Cambridge, UK, dilution 1:100) and was analyzed by fluorescence microscopy (immunoflurescence), while the second clone covers all p63 isoforms (pan-p63; clone DAK-p63) and was stained with standard ICC staining. Since the nuclear expression of the two p63 clones was highly correlated, immunohistochemical (IHC) staining was used in further analysis to demonstrate pan-p63-positive nuclei.

Cells from the culture suspension were spread (100 μL) on Super Frost positively charged slides, air-dried and kept at 4 °C until staining. Staining was performed according to a standard laboratory protocol in an automated Dako Autostainer Link48 instrument. Before ICC staining, each slide with cells was fixed in ice-cold acetone for four minutes and washed with DPBS. Membrane permeabilization was performed by heating in 10 mM citrate buffer for 20 min at 98 °C in a water bath. After cooling, the slides were transferred to the Autostainer for staining. Tissue on slides was treated with 3% hydrogen peroxide (5 min) to block endogenous peroxidases, followed by washing in DPBS and application of primary antibodies: p63 (DAK-p63, Dako, Denmark, dilution 1:100), Ki67 (MIB-1, Dako, Denmark, dilution 1:100) and CK3 (ab68260, Abcam, Cambridge, UK, dilution 1:50) for 45 min at room temperature. After repeated washings, a secondary antibody from EnVision FLEX, Dako, Denmark detection kit was applied for 45 min (K8010, Dako, Denmark) followed by washing and 3,3′-diaminobenzidine chromogen (DAB) for 10 min. Finally, the cells were counterstained with hematoxylin for two minutes, dehydrated, cleared, and cover slipped. ICC-stained cells were analyzed on a bright-field microscope (APX100 digital imaging system, Olympus, Japan), and microphotographs were taken using a digital camera (DP23, Olympus, Japan).

### Immunohistochemical staining

ICC and IHC staining protocols differ very little, mainly in the process of preparing cells or tissues for staining. After the cultivation of LSCs on the hAM, the membrane was fixed for 24 h in 4% buffered formalin. Then, the membrane was placed in cassettes, and the tissue was processed to paraffin by a routine fully automated process in histokinette. In short, the tissue was dehydrated through an increasing series of alcohols, purified in xylene-substitution, and immersed in melted paraffin. The whole process took 22 h, after which membranes with cells were embedded in paraffin blocks.

For further IHC analysis, several 3 µm cross-sections of tissue were cut from each paraffin-embedded block and dried in a thermostat at 65 °C for 1 h. One section was mounted on a glass slide and routinely stained with hematoxylin and eosin (H&E) for histological analysis. Other cross-sections were placed on positively charged immuno-slides and stained with the same primary antibodies as culture cells. Before IHC staining, antigen retrieval was performed in high pH 9.0 Tris(hydroxymethyl)aminomethane (TRIS) buffer at 97 °C for 20 min in PTLink (Dako, Denmark). The Autostainer Link48 automated instrument (Dako, Denmark) for IHC staining was used. From this point, the protocol for IHC staining is the same as described in the ICC section. The same anti-p63, anti-Ki67 and anti-CK3 primary antibodies as the same detection kit were used. IHC-stained cells were analyzed on a bright field microscope (ApexView APX100, Olympus, Japan), and microphotographs were taken using a digital camera (DP23, Olympus, Japan). After ICC and IHC staining, the percentage of p63 and Ki67 positive cells was determined in such a way that the number of positive and negative cells was recorded by counting more than 100 cells per sample. The size of LSCs grown in cell culture and on hAM was measured. For this purpose, a bright-field microscope (APX100 digital imaging system, Olympus, Japan) and digital camera were used (DP23, Olympus, Japan). The p63-stained cells were photographed under medium magnification of the microscope (200x) while defining the size of the objective in the program itself.

#### Flow cytometry

Flow cytometry (FCM) was used to determine the proportion of residual mouse 3T3 feeder cells in expanded LSC cultures and to analyze the expression of p63α and ABCG2 stem cell-associated markers. For the analysis of the remaining 3T3 feeder cells, the cell suspension was divided into two groups to determine nonspecific isotype antibody staining (rat IgG1 peridinyl chlorophyllin (PE)-conjugated antibody: BD Pharmingen, USA, dilution 1:100)) and specific anti-mouse feeder cell rat IgG1 PE antibody (Clone mEF-SK4, Miltenyi Biotech, Germany, dilution 1:100)). Unspecific staining was set to 1% of the acquired cells according to isotype antibody staining. Surface ABCG2 marker staining was performed similarly to the detection of 3T3 cells using an anti-ABCG2 allophycocyanin (APC) conjugated antibody (clone RD-FAB995A, R&D Systems, dilution 1:20), whereas intracellular localization of p63α required cell fixation and permeabilization steps. Briefly, cells were fixed in 4% PFA and permeabilized with 90% ice cold methanol. Upon washing in DPBS and blocking nonspecific staining with normal mouse IgG, fluorescein conjugated monoclonal antibody for p63α, human origin (clone c-12, Santa Cruz Biotechnology, Santa Cruz, CA, USA, dilution 1:60) was added. For each tube, 10,000 cells were acquired on a BD LSR II flow cytometer at CRKTB University of Immunology and analyzed using FlowJo software ver. 10.7.2 (Becton Dickinson, USA).

#### Droplet digital polymerase chain reaction analysis

DNA was isolated from the cell culture with the use of a QIAamp Blood Mini kit (Qiagen) according to the protocol for blood cells. Four different samples of LSCs were isolated and analysed. For the positive control for the mouse assay, DNA from the pure 3T3 cell line was used; 1 × 10^6^ cells were isolated. DNA isolated from human whole blood was used as the positive control for the human assay. For the purposes of quantification of mouse DNA, an assay was designed on chromosome 1 (GRCm39: 79,348,900–79,349,054); for human DNA, the assay was designed on chromosome 7 (GRCh38.p13: 5,562,562–5,562,812) as described in Prosenc Zmrzljak et al. [26].

Each 20 µL droplet digital polymerase chain reaction (ddPCR) consisted of 10 µL of ddPCR Supermix for Probes (Bio-Rad, USA), 1 µL of Hind III (New England Biolabs, USA) and 4 µL of DNA. For assessment of the human DNA background, the DNA was diluted 10 × for use in the ddPCR mixture. The reaction mixture was prepared as stated in Prosenc Zmrzljak et al., 2021 [26]. The cycling conditions specific for this assay were as follows: 95 °C 10 min, 1 cycle; 94 °C 30 s, 58 °C 1 min, 40 cycles; 98 °C 10 min, 4 °C ∞. All samples had droplet counts higher than 12,000. Samples were measured with the mouse assay in four replicates for each sample and with the human assay in one replicate. The potential cross-reactivity was tested on DNA isolated from pure mouse or human samples.

#### Immunofluorescence, imaging and analysis

For all protocols presented in this section, LSCs were seeded in uncoated glass microwell 35 mm dishes with 0.16–0.19 mm glass thickness (#1.5 coverglass; MatTek Corporation, Ashland, MA, USA) and maintained until the time of the experiment at 37 °C and 5% CO_2_ in a Galaxy 170 s humidified incubator (Eppendorf, Hamburg, Germany). LSC colonies analyzed in experiments presented in this section were from different passages (zero and first), while 3T3 feeder cells were treated with mitomycin C.

To visualize α-tubulin, cells were fixed with a microtubule-preserving mixture of 4% PFA and 0.25% glutaraldehyde (GA) for 10 min at room temperature. To visualize p63, cells were fixed in 4% PFA solution only. Fixed cells were then washed with 1 mL of DPBS three times for five minutes. Next, permeabilization was performed in 0.5% Triton-X-100 in DPBS for 15 min. To block nonspecific binding of antibodies, cells were incubated in 500 mL of blocking buffer, 1% normal goat serum (NGS), for 1 h at 4 °C. Cells were then incubated in 500 mL of primary antibody in 1% NGS solution for 24 h at 4 °C. The following primary antibodies were used: rat monoclonal anti-alpha Tubulin YL1/2 (MA1-80,017, Invitrogen, CA, SAD, dilution 1:300), rabbit monoclonal anti-p63-α (D2K8X XP, Cell Signaling Technology, dilution 1:100) and mouse monoclonal anti-p63 [4A4] (ab735, Abcam, Cambridge, UK, dilution 1:100). After the primary antibody, cells were washed in DPBS and then incubated in 500 mL of secondary antibody in 1% NGS solution for 1 h at room temperature. The following secondary antibodies were used: donkey anti-mouse IgG Alexa Fluor 488 (ab150112, Abcam, Cambridge, UK), donkey anti-rabbit IgG Alexa Fluor 647 (ab150063, Abcam, Cambridge, UK), and donkey anti-rat IgG Alexa Fluor 594 (ab150156, Abcam), all diluted 1:250. Finally, cells were washed with 1 mL of DPBS three times for 5 min. For actin staining, SiR-actin dye (Spirochrome, 100 nM, added 15 min before imaging) was used, and for DNA staining, 1 mg/mL 4′,6-diamidino-2-phenylindole (DAPI) solution (added 15 min before imaging) at a 1:1000 dilution in DPBS was used.

Most live cells and, when stated, fixed cells were imaged using the spinning disk confocal microscope system (Dragonfly, Andor Technology, Belfast, UK) equipped with a 63x/1.47NA HC PL APO oil objective (Leica) and Zyla 4.2P scientific complementary metal oxide semiconductor (sCMOS) camera (Andor Technologies). During imaging, cells were maintained at 37 °C and 5% CO_2_ within an H301-T heating chamber (Okolab). Images were acquired using Fusion software (v 2.2.0.38). For excitation, 405-nm and 640-nm laser lines were used for visualization of blue and far-red fluorescence, respectively. For live cell images, up to 20 z-planes were acquired and imaged sequentially with both laser lines every 1 min with a 150 ms exposure time for 30 min. The following live-cell dyes were used: NucBlue-Hoechst 33,342 (Invitrogen, R37605, added immediately before imaging) and SiR-actin (Spirochrome, 100 nM, added at least 3 h before the start of imaging).

Fixed cells, unless otherwise stated, were imaged using a Bruker Opterra I multipoint scanning confocal microscope (Bruker Nano Surfaces, Middleton, WI, USA). In experiments in which the whole spindle stack was imaged, z-stacks were acquired at 30–60 focal planes for immunofluorescence images, separated by 0.5 μm with unidirectional xyz scan mode. A 60 mm pinhole aperture was used, and the xy-pixel size was 83 nm. For excitation of blue (DAPI), green (anti-p63 conjugated to a secondary antibody with a green AlexaFluor488 dye), red (anti-a-tubulin conjugated to a secondary antibody with a red AlexaFluor594 dye) and far-red fluorescence (SiR-actin) dyes, 405, 488, 594, and 647 nm diode laser lines were used, respectively. Excitation light was separated from the emitted fluorescence using the dichroic and barrier filter set for 405/488/561/640 nm (DAPI/eGFP/TRITC/Cy5) (Chroma, USA). Images were captured with an Evolve 512 Delta EMCCD camera (Photometrics, Tucson, AZ, USA) using a 300 ms exposure time and without binning. The frame average was performed eight times for immunofluorescence images. All experiments were carried out using a Nikon CFI Plan Apo VC 3 100x/1.4NA oil objective (Nikon). The system was controlled with Prairie View Imaging Software (Bruker Nano Surfaces).

In experiments for the comparison of p63α and p634A4 (recognizing all p63 isoforms) signaling, fixed cells were imaged using a Zeiss Airyscan LSM 800 confocal microscope with Axio Observer. Z1 inverted stand (Carl Zeiss GmbH, Germany). Z-stacks were acquired with six focal planes separated by 1 μm. For excitation of blue, green, red and far-red fluorescence, 405/488/561/640 laser lines were used, respectively. For DAPI/AF488/AF594/AF647 intensity detection, 41 μm, 45 μm, 51 μm and 56 μm pinholes were used, respectively, with 200 nm xy pixel size. Images were captured with an LSM 800 camera using a 1.03 μs exposure time, without binning and no frame averaging. Images were taken in the tiles module with Plan-Apochromat 63x/1.40 Oil DIC M27 objective (Carl Zeiss GmbH, Germany). The system was controlled with ZEN blue 3.4 system software (Carl Zeiss GmbH, Germany).

#### Microbiological control

Since limbal grafts cannot be subjected to terminal sterilization and due to a high risk of microbial contamination in all processing steps, it is extremely important to apply prescribed measures to prevent contamination in all operations, i.e., procurement, preparation, preservation, and packaging of cells. Microbiological control of samples obtained in the processing of LSCs for the presence of anaerobic and aerobic bacteria and fungi was carried out according to the Guide to the quality and safety of tissues and cells for human application published by the European Directorate for the Quality of Medicines & HealthCare of the Council of Europe (EDQM). Appropriate microbiological tests were carried out, the eye swab of the donor was taken as a starting point, then the eye swab before the transplant itself, the transport medium was analyzed, and the environment was monitored during the aseptic preparation of the limbal grafts. Additionally, the following samples were microbiologically tested: 10% limbal biopsy washing medium with 5% penicillin‒streptomycin-amphotericin B, medium during cell growth in p0 and P1, hAM swab after thawing that was used as a scaffold for growing LSCs, and cell medium just before clinical use during the final packaging of the graft. Briefly, the sterility test was carried out under aseptic conditions by a direct inoculation method suitable for solutions, tissue samples and swab heads. Thioglycollate broth with resazurin (Biomerieux, Craponne, France) was used for cultivation of anaerobic bacteria at 37 °C, and soya-bean casein digest medium (Biomerieux, Craponne, France) was used for the culture of both fungi and aerobic bacteria at 22 °C. The quantity of the test sample was transferred into the culture medium so that the volume of the product did not exceed 10% of the volume of the medium. When the test sample had antimicrobial activity, the test was carried out with appropriate neutralizing substances. The inoculated media were incubated for 14 days. The described microbiological tests were carried out in an accredited contract laboratory of the Croatian Institute for Transfusion Medicine, Zagreb, Croatia.

#### Detection of bacterial endotoxins

Detection of bacterial endotoxins was performed using a Lysate of Amebocyte Limulus test (LAL) that responds to lipopolysaccharide (LPS) as previously described Hochstein, H. D [27, 28]. Endotoxins were measured in endotoxin units per milliliter (EU/mL). One EU is approximately 0.1 to 0.2 ng of endotoxin/mL solution. The LAL kinetic chromogenic test can detect as little as 0.005 EU/mL endotoxin (method D, European Pharmacopeia. 2.6.14) [29] using the Endosafe®nexgen-PTS™ device (Charles River, SC, USA). This device is an automated and portable spectrophotometer approved by the Food and Drug Administration (FDA) and allows fast, automatic analysis, data processing and printing of results on an attached small printer. Single-use Endosafe® PTS Cartridges (Charles River Laboratories, Charleston, SC, USA) with a sensitivity of 0.5 to 0.005 EU/mL and a dilution of 1:25 were used for the analysis. The method has been characterized as highly sensitive and highly specific and is particularly reliable in terms of avoiding false negative or false positive results. Quantification of bacterial endotoxins in the limbal graft on the hAM was performed at the end of cultivation, i.e., before clinical application. The maximum dilution valid (MDV) was calculated by the formula:

$$MDV=\frac{\mathrm{EL\, x\, concentration\, of\, test\, solution}}{\uplambda }$$

The test solution concentration value was equal to 1.

All samples (n = 6) were prepared in sterile disposable pyrogen-free plastic bottles.

Sample preparation: From a total of 3–4 mL of test sample (cultured medium GM, FM or L-15 medium), 400 µL (stock solution) was used in the procedure. When preparing a 1:10 dilution, the test sample (400 μL) was diluted in 3600 μL of LAL water. For a further 1:25 dilution, 1600 µL from tube 1 was added to tube 2 with 2400 μL of LAL water. PTS cartridges have four channels: two of the four channels contain endotoxin spikes and LAL (used as the positive product control), and the other two contain only LAL (for testing of samples). After entering the basic information in the PTS device (operator ID, cartridge lot, cartridge calibration code, sample lot, sample ID and dilution factor), the test sample from test tube 2 was loaded into the four wells of the cartridge, 25 μL in each. After that, the enter key was pressed, and the pump on the device drew samples from all four wells. For complete analysis of one sample, approximately 15 min was needed.

#### Statistics, quantification and analysis

Data are presented as the mean ± standard deviation (SD) of the mean. Cell size was measured in ImageJ/Fiji (National Institutes of Health, https://imagej.nih.gov/ij) [30] by using the ‘Polygon selection’ tool in the channel where actin was labeled as the extent of the actin signal and was used as a proxy for the cellular boundary. The size of the nucleus was measured by the same method in the channel where DNA was labeled as the extent of the DNA signal and was used as a proxy for the nuclear boundary. The signal was measured across single z-planes to avoid mistakes due to cell overlapping in multilayered structures of the stem cell colonies. The average fluorescence intensity signal of p63 was measured in a single cell by using the ImageJ ‘Polygon selection’ tool on the sum-intensity projection of all acquired z-planes. The cell boundaries were identified by the extent of the SiR-actin signal, while the average p63 signal was measured in the p63 channel. The background fluorescence intensity measured in the cytoplasm by using a 1 × 1 µm rectangle was subtracted from the values obtained, and the calculated integrated intensity value was divided by the number of z-stacks used to generate the sum projection of each cell. Colony area was measured on individual colonies that included ‘small’ and mostly p63 positive cells where the boundary of a colony was defined by the start of a strong signal of neighboring mouse 3T3 feeder cells. Colonies with less than ∼10 cells were excluded from the analysis. Spindle length was measured in ImageJ as the distance between spindle poles in immunofluorescence images of mitotic spindles where spindle poles were discerned as the center of a strong tubulin signal (see Fig. 4). Spindle width was measured in ImageJ as the distance between the two outermost observable k-fibers on immunofluorescence images of mitotic spindles by using a line perpendicular to the main spindle axis defined by the spindle pole-pole axis (see Fig. 4). Quantification and data analysis were performed in R (R Foundation for Statistical Computing). Figures and schemes were assembled in Adobe Illustrator CC (Adobe Systems). Statistical analysis was performed using the two proportions z test.

## Results

### Characterization of limbal stem cells in 2D culture

#### Potency (yield, viability, population doubling time, colony-forming efficiency, p63, Ki67)

In order to evaluate the potential of cultured limbal grafts, identity and potency (the yield of cells dissociated from the 2 mm^2^ limbus biopsy, viability, population doubling time, colony-forming efficiency as well as percentage of holoclones, meroclones and paraclones) were investigated.

**Fig. 2:**
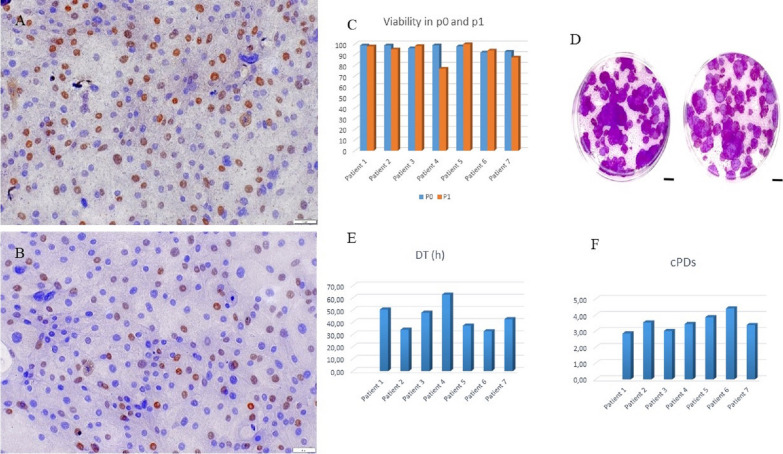
Evaluation of potency of LSC in 2D cultures. **A** Positively brown stained nucleus of LSCs for p63 and **B** Ki67 antibodies specific to human cells shown by the immunocytochemistry (ICC) method. The staining method was conducted at the end of the cultivation period when LSCs dominated the culture and confluence exceeded 90% (magnification 100×). **C** Viability of LSCs in p0 and p1; **D** For the Colony-Forming Efficiency (CFE) assay, which was conducted in duplicate for three donors, we assessed the proliferation and differentiation capacity of 1000–1500 seeded limbal stem cells (LSCs) cultured on 3T3 irradiated feeder layers in Petri dishes. After 14 days of culture, a total of 86 ± 13.61 cell colonies were observed, resulting in a calculated CFE of 7.08 ± 1.55%. Data are presented as mean ± SD of all samples. Scale bar 10 mm; **E** Population doubling time (DT) in hours and **F** cumulative population doubling (cPDs) were determined for each patient and are represented in histograms

After three-step enzymatic dissociation, the yield from the first tube was 70 234 ± 39 cells, (seeded approximately 23–25 000 into 6-well tissue culture plates, in three fields) and labeled 1/1; 1/2; 1/3; from the second tube it was 34 531 ± 14, and in the third 16 625 ± 14 (labeled as 2/1 and 3/1). The yield, represented as the mean ± SD of all samples, following the enzymatic digestion of LSCs from the 2 mm^2^ limbus biopsy specimen, was 93 203 ± 37. The percentage of positively stained cells for p63 was 71.0 ± 11.3% and for Ki67, it was 41.0 ± 12.6% (Fig. 2A, B).

When LSCs in primary cell culture reached approximately 75–80% confluence (assessed visually), the estimated viability of p0 was 96.42 ± 2.88%, and for p1, it was 93.19 ± 8.08% (Fig. 2C). From the initially seeded 1.44 × 10^5^ cells in p1, 1.73 × 10^6^ ± 6.86 LSCs grew when the cell cultures were confluent. The CFE results obtained for three donors in duplicate were 7.08 ± 1.55% (Fig. 2D). Additionally, percentages of holoclones (10.10 ± 3.86%), meroclones (80.94 ± 3.2%), and paraclones (9.48 ± 0.42%) were determined, reflecting the differentiation states of the colonies. The average time of limbal cell culture in p1 until reaching ideal confluence for tissue engineered limbal grafts was six days. The DT was calculated as 1.83 days, equivalent to 43.92 h, while the cPDs stood at 3.50 (Fig. 2E, F), presented as the mean ± SD of all samples. LSC form 2D colonies analyzed in p1 seeded at a concentration of 1000 or 1500 cells per Petri dish 100 with previously prepared feeder cells (1 × 10^6^ 3T3 cells treated with mitomycin C). More p63-positive cells with a lower percentage of Ki67 proliferation were observed in p0, while p1 cells showed a higher percentage of proliferation. LSCs in single-cell suspension are not suitable for size measurement since they are adherent cells. It is acceptable to measure cell size or surface area in 2D or 3D cell culture. Therefore, it was decided that before ICC staining, the cells should be seeded on glass slides for less than 24 h so that they adhere to the surface. In this case, larger and more transparent cells are obtained for morphological and ICC analysis.

### Identity, morphology, cell and nuclear size of limbal stem cells

**Fig. 3:**
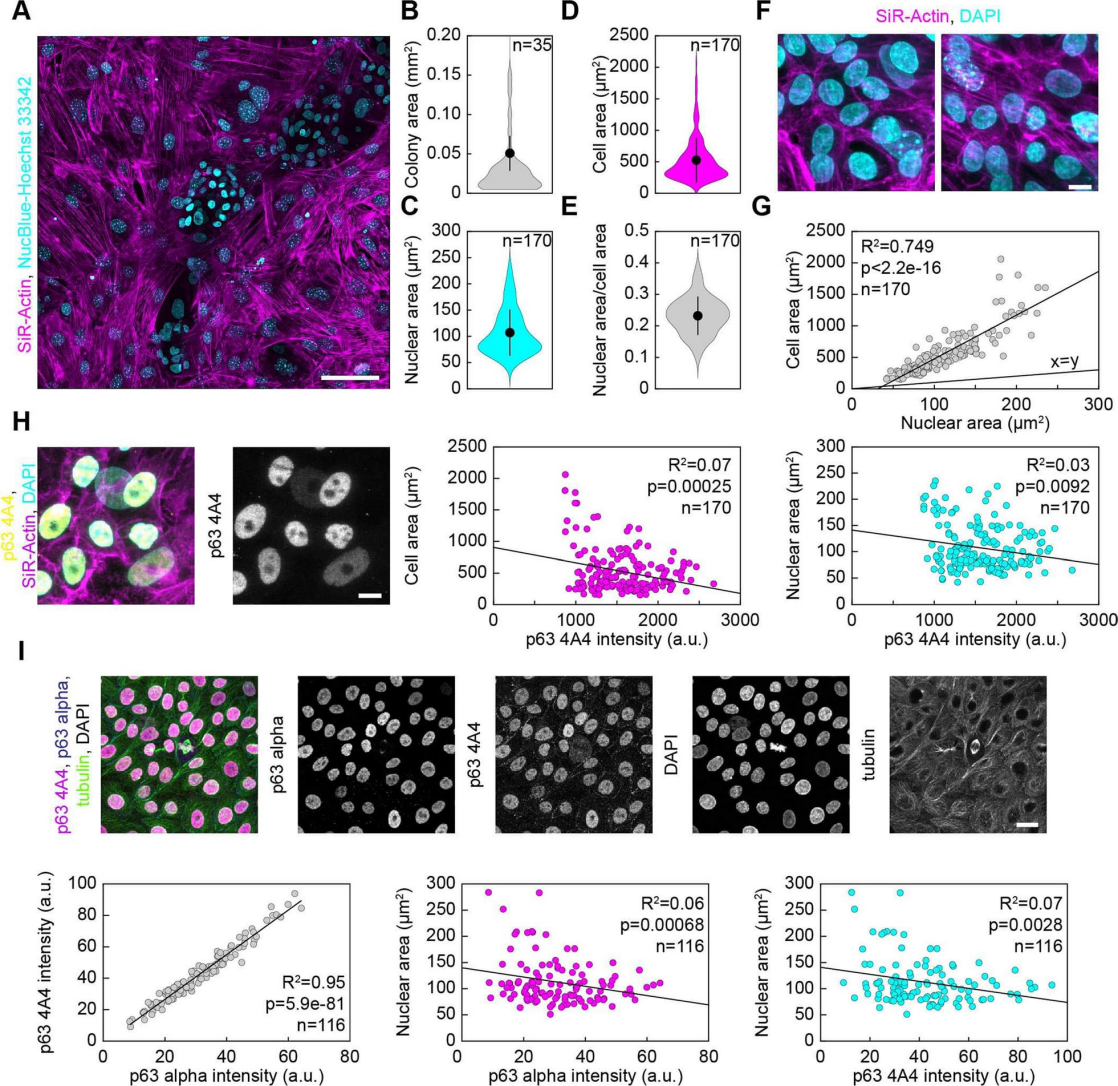
Colony, cell, and nuclear area of LSCs and relation of morphological parameters to p63 levels. **A** Fluorescence image of live LSC colonies (small cyan nuclei within cells very weakly stained with SiR-actin, magenta) growing within 3T3 feeder fibroblasts (much bigger cyan nuclei with larger nucleoli dots within nucleus, seen as intense cyan dots, within cells that are strongly stained for SiR-actin showing actin filaments, magenta). Cyan, NucBlue-Hoesht-33342; magenta, SiR-actin. Maximal projection of multiple z-stacks. **B** Area of colonies of LSCs, black dots, mean; black lines, standard deviations. **C** Nuclear area of individual LSCs within colonies, **D** Cell area of individual LSCs within colonies, **E** Nuclear area divided by cell area (nuclear to cytoplasm ratio, N/C) of each individual LSC within colonies. **F** Immunofluorescence image of fixed LSCs within the colony stained with SiR-actin (actin, magenta) and DAPI (DNA, cyan). Maximal projection of multiple z-stacks. **G** Nuclear area versus cell area across the population of LSCs within colonies. The black line represents the linear regression fit, or x = y line. **H** Immunofluorescence image of fixed LSCs within the colony stained with SiR-actin (actin, magenta), DAPI (DNA, cyan), and secondary antibody AlexaFluor488 conjugated to primary antibody raised against p63 4A4 (yellow). Maximal projection of multiple z-stacks. (left), p63 intensity in each cell versus its total area (middle), or its nuclear area (right). Black lines in (**H**) represent linear regression fit. **I** Immunofluorescence image of LSCs within the colony stained with secondary antibody AF647 conjugated to primary antibody raised against p63-α (dark blue), secondary antibody AF488 conjugated to primary antibody raised against p63 4A4 (magenta), DAPI (DNA, gray), and secondary antibody AF594 conjugated to primary antibody raised against tubulin (green). Maximal projection of multiple z-stacks. (left) p63-α versus p63 4A4 signal intensity in each cell; p63-α (middle) and p63 4A4 (right) intensity versus their nuclear area. Black lines represent linear regression fit

After performing potency analyses on LSC, our objective was to visualize colonies of LSC in their in vitro culture environment. To visualize limbal cell colonies in their in vitro cultured state and at superior resolution, stem cell colonies seeded on mouse 3T3 feeder cells were fixed and stained with SiR-Actin (actin filament dye) [31] and DAPI (DNA dye) or live-imaged cells in the presence of SiR-Actin and NucBlue-Hoechst (DNA dye). Then, multiple whole fields of view were imaged in 3D (x, y, z) by fluorescence confocal microscopy. Interestingly, it was observed that the SiR-actin signal was much stronger in mouse feeder 3T3 cells than in LSC organized in colonies (Fig. 3A), enabling easy visualization of stem cell colonies. Stem cells in colonies were further characterized by much smaller nuclear sizes (Fig. 3C) and distinctive nuclear morphologies with a homogeneous DAPI signal (Fig. 3H) when compared to 3T3 feeder cells. Multiple dispersed colonies of stem cells incorporated between 3T3 cells were detected, mostly of regular circular or extended shape (Fig. 3F). The sizes of LSC colonies were variable but averaged 0.05 ± 0.02 mm^2^ (n = 35 colonies, six fields of view). Furthermore, the basic cell and nuclear parameters of LSC within the colonies in their cultured in vitro state were quantified by measuring the extent of cortical actin signal as a proxy for cell size and the extent of DNA signal as a proxy for nuclear size. The average cell area of LSC was 524.87 ± 26.51 μm^2^ (n = 170 cells), and the average nuclear area was 107.07 ± 3.30 μm^2^ (n = 170 nuclei), which gave an average nuclear/cell area ratio (N/C) of 0.231 ± 0.004 (Fig. 3B–E). Then, a comparison was made to ascertain how the size of the nucleus related to the size of the LSC across the population of cells in colonies. A strong positive correlation (R^2^ = 0.749, p = 2.2e^−16^) was observed between the nuclear and cell size of individual stem cells, implying that the nuclear size scaled strongly with the cell size across the population of cells, as expected from the previous data on multiple types of human cells [32].

To explore the relationship between cell and nuclear size and p63 status (marker of stemness) of each individual cell, stem cell colonies were labeled with SiR-actin, DAPI and p63-specific primary antibodies conjugated with secondary antibodies coupled to a red dye (see Methods), and multiple fields of view of LSC colonies were imaged in 3D by fluorescence confocal microscopy. The average level of p63 in every cell was quantified by calculating the average intensity of the p63 signal in summed projections of imaged z-planes in each individual cell (see Methods). The average level of p63 in every cell was then compared with basic cell morphological parameters, measured as described above. All the inspected interphase cells were positive for p63, and the level of p63 within the cell was inversely correlated with the size of the cell (Fig. 3H, R^2^ = 0.07, p = 0.00025) and the size of its nucleus (Fig. 3H, R^2 ^= 0.03, p = 0.0092, Table 1). These results imply that the p63 level, and thus the stemness of individual cells, decreased with increasing nuclear and cell size. Therefore, these results suggest that cells within the imaged population had variable stem cell potential (see Discussion).

**Table 1:** New characterization and safety evaluation of LSCs in 2D culture at p1 by fluorescence confocal microscopy

Parameter	No. of analyzed cells	Value/range
ΔNp63α and p63 (4A4)^+^ cells	116	100%*
Cell area of p63^+^ cells	170	524.87 ± 26.51 μm^2^
N/C ratio	170	0.231 ± 0.004
Mitotic spindles (length)	12	11.40 ± 0.54 μm
Mitotic spindles (width)	12	8.05 ± 0.55 μm
Aberrant interphase cells	873	< 2.5%
Multipolar phenotype during metaphase	47	2.12 ± 2.10%
Chromosome bridge or lagging chromosome phenotype during anaphase	26	3.84 ± 3.77%

^*^100% of the LSC that stained positive for ΔNp63α were also positive for p63 (4A4) cells

The p63 4A4 monoclonal antibody recognizes several isoforms in addition to the truncated ΔNp63α form. The α is the isoform of ΔNp63 essential for regenerative proliferation of LSCs; therefore, to confirm the high percentage of stemness and the specificity obtained from the p63 signal, p63 4A4 immunostaining was repeated together with a p63-α antibody that recognizes only the α-subunit of p63 (Fig. 3I). All interphase cells were positive for both p63 clones, and their signal intensities were strongly correlated (Fig. 3I, R^2^ = 0.95, p = 5,9e^−81^). The signal intensity of both clones inversely correlated with their nuclear size (Fig. 3I, R^2 ^= 0.07, p = 0.0006 for the p63α form, R^2^ = 0.07 p = 0.0028 for the p63 4A4 form), Table 1.

### Safety evaluation

#### Mitotic spindle parameters in limbal stem cells

**Fig. 4:**
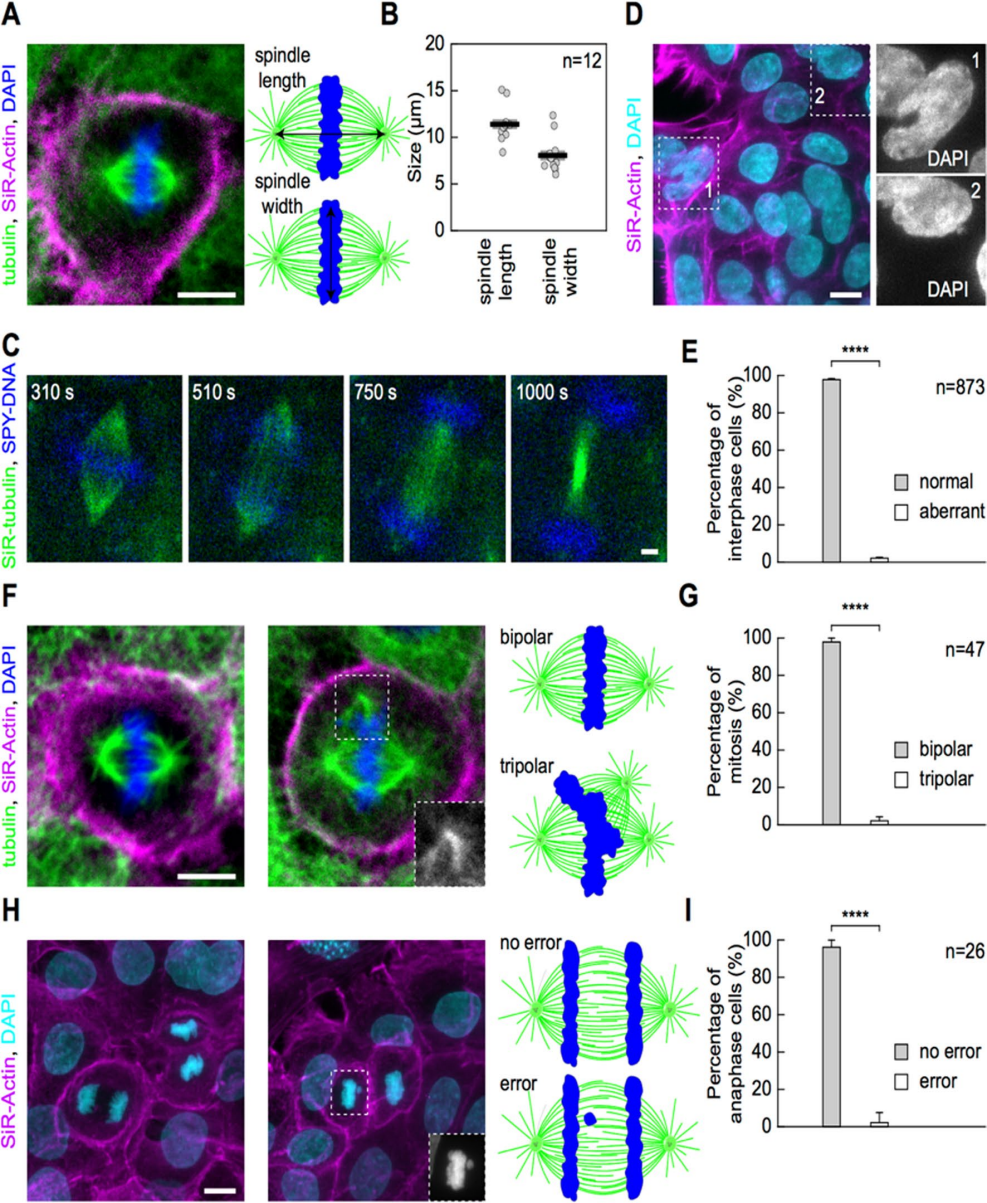
Mitotic spindle morphology in live-imaged and fixed LSCs and mitotic fidelity parameters. **A** Immunofluorescence image of an LSC during metaphase of mitosis. Blue, DAPI; magenta, SiR-actin; green, secondary antibody AlexaFluor594 conjugated to primary antibody raised against α-tubulin. Maximal projection of multiple z-stacks. **B** Schematic representations of metaphase mitotic spindles showing how spindle length and width were measured from immunofluorescence images (left) and measurements of spindle length and width across metaphase spindles in LSC grown in colonies (right). **C** Time frames of live LSC during mitosis from metaphase to telophase (left to right). Blue, SPY-DNA, green, SiR-tubulin. **D** Immunofluorescence image of fixed LSCs within the colony stained with SiR-actin (actin, magenta) and DAPI (DNA, cyan). Maximal projection of multiple z-stacks. Insets (gray, DAPI) show abnormal nuclear morphology (top) and the presence of micronucleus (bottom) in interphase LSCs presented on the left. **E** Percentage of LSCs with normal and aberrant interphase nucleus. **F** Immunofluorescence images of fixed LSCs within the colony during metaphase of mitosis stained with SiR-actin (actin, magenta), DAPI (DNA, blue), and secondary antibody AlexaFluor594 conjugated to primary antibody raised against α-tubulin (microtubules, green). Maximal projection of multiple z-stacks. The left cell has two spindle poles (bipolar), while the right cell has three spindle poles (tripolar), where the additional pole is shown within the inset (gray, microtubules). **G** Schematic representations of metaphase mitotic spindles showing how bipolar and tripolar spindles were defined (left) and percentages of LSCs with bipolar or tripolar spindles during metaphase (right). **H** Immunofluorescence images of fixed LSCs within the colony during anaphase/telophase of mitosis stained with SiR-actin (actin, magenta), DAPI (DNA, cyan) with insets (gray, DNA) representing chromosomes separated from the main mass of chromosome during anaphase (left), schematic representations of anaphase/telophase cells showing how ‘no error’ and ‘error’ categories of anaphase/telophase cells were defined (middle), and quantification of percentage of anaphase/telophase cells with error and no error phenotypes

To our knowledge, there have been no data on mitotic spindles in LSCs, and it was decided to quantify the basic parameters of mitotic spindle morphology in fixed unsynchronized stem cells in their cultured state in vitro. Stem cells in culture were labeled with SiR-actin, DAPI and α-tubulin-specific primary antibodies conjugated with secondary antibody coupled to a green dye (see Methods), and multiple fields of view of LSC colonies were imaged in 3D by fluorescence confocal microscopy. Then, metaphase spindle length was measured as the distance between spindle poles based on the tubulin signal (Fig. 4A), and spindle width as the distance between the outermost kinetochore fibers within the mitotic spindle (Fig. 4B), based on the same signal. The spindle length was on average 11.40 ± 0.54 μm, and the spindle width was 8.05 ± 0.55 μm (Fig. 4B), thus demonstrating a rather elongated spindle shape when compared to human differentiated cells in culture, especially those of tumor origin [33]. Spindle length and width were correlated in the LSCs during metaphase (R^2^ = 0.8653, P = 7 × 10^–6^). Live-cell imaging of LSCs in culture was also performed by labeling cells with SiR-tubulin (microtubule dye) and SPY-DNA (DNA dye) [31] and recording whole z-stacks of LSCs in 4D (x, y, z, t) by fluorescence confocal microscopy (see Methods). Metaphase spindles were observed to be of similar shape to those observed by imaging of fixed cells (Fig. 4A, F), which successfully entered anaphase, divided duplicated chromosomes into two newly forming daughter cells, and initiated cytokinesis without any apparent mitotic defects.

### Proportion of erroneous cell divisions in limbal stem cell colonies

To estimate the proportion of mitotic defects that can lead to various cell cycle and growth abnormalities (including aneuploidy and micronuclei formation) [34] in a larger population of LSCs than could be achieved by a live-cell imaging approach, the number of mitotic and mitotic-related defects was scored in a fixed population of LSCs. First, the presence of nuclear aberrations that are associated with mitotic defects was checked, including abnormal shapes of interphase nuclei with a lobed appearance (Fig. 4D), the presence of micronuclei (Fig. 4H), and the presence of binucleated cells (Fig. 4D) in the colonies of LSC.

These nuclear abnormalities are often preceded by mitotic defects such as lagging chromosomes, chromosome bridges, unaligned chromosomes, or complete malfunction of cytokinesis but can also be related to defective DNA replication that occurs during the preceding S phase [35]. By scoring large numbers of interphase cells (n = 873), only a small fraction (< 2.5%) of aberrant interphase cells was observed in a population of LSC within colonies. To characterize in detail the proportion of cells with mitotic defects in these colonies, the number of metaphase cells was scored with multipolar mitotic spindles as judged by the tubulin signal (Fig. 4F, G), and anaphase/telophase cells with a chromosome bridge/lagging chromosome phenotype as judged by the DNA signal within the spindle midzone that was clearly separated from the main mass of chromosomes (Fig. 4H, I). Regarding the prevalence of spindle defects, 2.12 ± 2.10% of mitotic spindles exhibited a multipolar phenotype during metaphase (Fig. 4G), and 3.84 ± 3.77% of anaphase cells had a DNA signal within the spindle midzone (Fig. 4I), indicating a chromosome bridge or lagging chromosome phenotype. This suggests that mitotic aberrations are rare in LSC culture.

### Characterization of limbal cells in a single-cell suspension

#### Identity and impurity

**Fig. 5:**
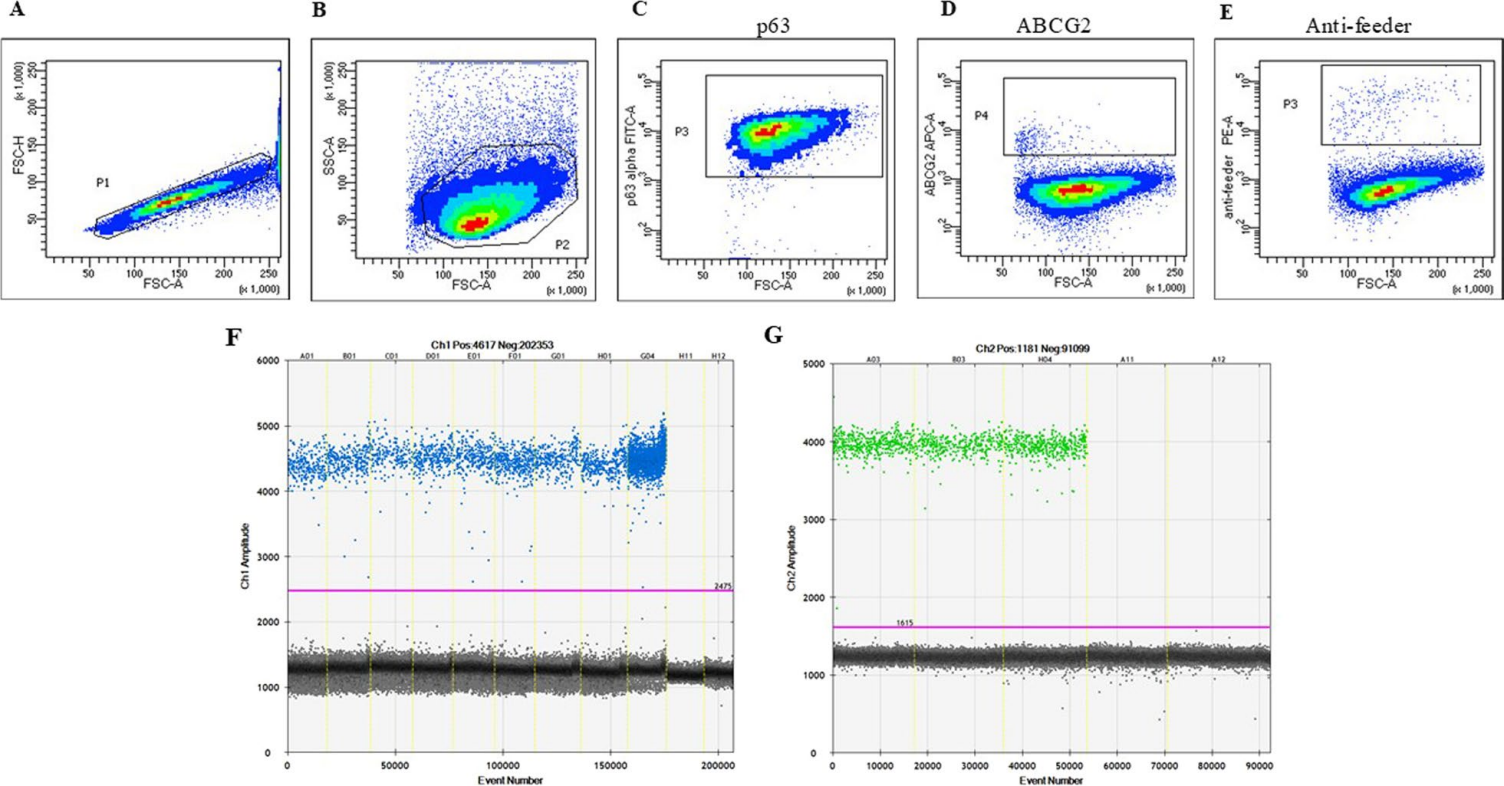
Representative flow cytometry and ddPCR analysis of LSCs and 3T3 cells prepared as the feeder layer. **A** and **B** Gating strategy of single-cell LSCs based on forward scatter (FSC) and side scatter (SSC) parameters. Unspecific staining was set to 1% of the acquired cells according to isotype antibody staining. **C** The majority of LSCs expressed p63α, **D** but few were positive for the ABCG2 stem cell-associated marker. **E** Residual 3T3 feeder cells in LSC expanded culture were below the acceptance limit. **F** 1D plot for two sets of analyses on ddPCR analysis of sample 3 (A1 – D1) and sample 4 (E1 – H1) with mouse assay. G4 is analysis of mouse positive control (DNA from pure 3T3 culture), H11 is negative control (NTC) and H12 is test for possible cross-reactivity with human DNA (human positive control). **G** Analysis of sample 3 (A3) and sample 4 (B3) with the human assay. H4 is analysis of human positive control (DNA from human whole blood), A11 is NTC and A12 is test for possible cross-reactivity with mouse DNA (mouse positive control)

Next, the expression of stem cell-associated markers p63α and ABCG2 was determined in LSCs in p1 after six days of cultivation on 3T3 feeder layer cells (the gold standard feeder layer for CLET) trypsinized and processed into a single-cell suspension. Validated FCM analysis showed that almost all LSCs expressed p63α (96.8% ± 0.837), while less than 1% were positive for ABCG2 (0.68% ± 0.231) (Fig. 5C, D). The residual growth-arrested 3T3 cells in LSC cultures were well below the acceptance criteria of 5% (1.76% ± 0.241) (Fig. 5E). Concerns related to the use of 3T3 feeder cells and the safety of cell therapy have existed for a long time, so this research was designed not only to monitor the percentage of residual feeder cells by FCM but also to demonstrate with another laboratory method such as ddPCR the reliability of the measurement as well as the advantages or disadvantages. In addition, four samples of LSCs cultivated on 3T3 feeder cells were tested in parallel to compare FCM and ddPCR results. These samples were derived from LSCs not intended for clinical use. For ddPCR, DNA was isolated from 6.87 × 10^5^ to 1.3 × 10^6^ total cells (Table 2). Since both assays (human and mouse) were designed on regions of the genomes where two copies are present per diploid genome, the ratio between mouse and human cells could be directly calculated. Each of the four samples was measured four times, and the average percentage of mouse 3T3 feeding cells was calculated in the human cell background. The DNA ddPCR determined these rates of 3T3 contents per sample: sample 1 (0.577%; CI 95% ± 0.082%), sample 2 (0.879%; CI 95% ± 0.132%), sample 3 (6.849%; CI 95% ± 0.357%) and sample 4 (1.024%; CI 95% ± 0.061%).

**Table 2:** Isolation of DNA from LSC cultures and analysis of residual 3T3 cells by comparison of two methods

Samples	No. of cells	Concentration [ng/uL]	ddPCR % residual 3T3 cells	FCM % residual 3T3 cells
Sample 1	1.3 × 10^6^	27.900	0.577	2.50
Sample 2	1 × 10^6^	23.950	0.879	1.20
Sample 3	4 × 10^5^	14.950	6.849	6.50
Sample 4	6.87 × 10^5^	28.000	1.024	1.90

### Characterization of tissue-engineered limbal grafts

#### Identity, cell size, morphology

**Fig. 6:**
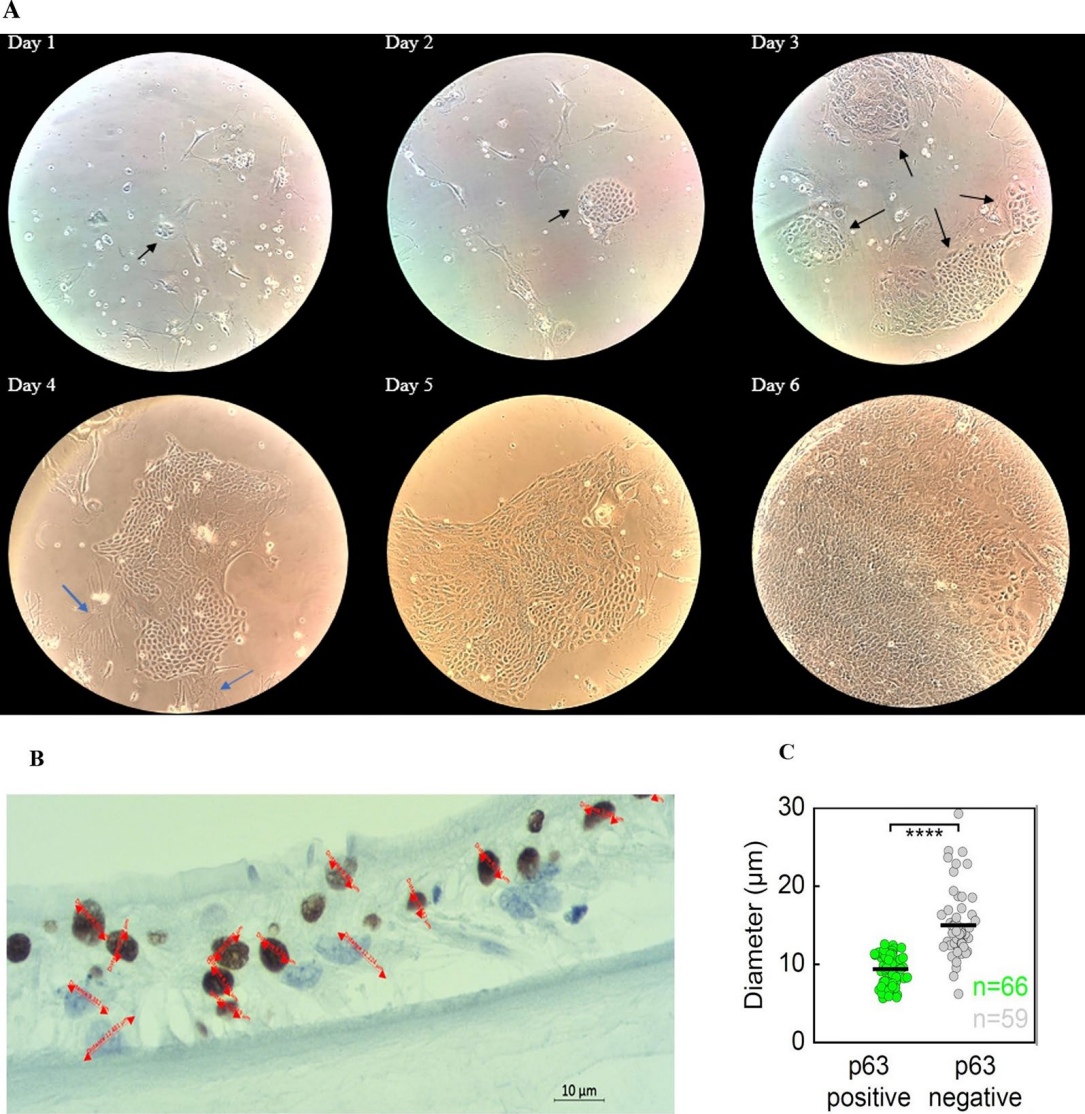
Evaluation of limbal graft. **A** To estimate the exact time when the graft was ready for clinical use, the growth of limbal cells in the control sample on the plastic surface was monitored daily. On the first day of culture, a small colony (approximately 5 cells) of limbal stem cells (LSCs) was cocultured with a feeder layer showing undifferentiated small-sized polygonal cells with little cytoplasm (arrows). They were surrounded by 3T3 feeder layer cells, which represent larger cells, elongated irregularly shaped (branched) fibroblasts. On the second day of cultivation, a nearly circular colony of LSCs with a smooth perimeter was visible. On the third day of cultivation, circular colonies joined together (arrows), expanded and pushed away the feeder cells. On the fourth, fifth and sixth day of culture, round colonies continued to grow, forming stratifications and merged with each other until confluence with a progressively decreasing amount of feeder cells. The results of measuring the size of LSCs grown in limbal grafts (**B**, **C**) that were p63-positive cells were significantly smaller than p63-negative cells (P < 0.001) (magnification, 100 ×)

The growth of limbal cells on the hAM cannot be monitored with an inverted microscope due to the structure of the membrane itself. For this reason, LSCs were seeded in the same number on a 6-well culture dish (1.44 × 10^5^) but without ring CellCrown™6 and hAM. At the same time, a smaller limbal graft on ring CellCrown™12 (with inner surface area 1.54 cm^2^) was placed into a 12-well culture plate that was prepared for histopathological analysis as part of quality control. Therefore, the growth of LSCs in the control sample was monitored daily with an inverted microscope, including how they merged into colonies filling the surface and pushing feeder cells away (Fig. 6A). That is, when LSCs reached subconfluency, which is usually after an additional six days of growth, and feeder cells are visible only in traces (< 2%), the limbal graft is ready for clinical use. The total cultivation time from biopsy to cultivation of two limbal grafts for each patient was 14 to 20 days. Measurement of cells cultured on the hAM showed that the average size of p63-positive cells was 9.4 ± 1.91 µm (median 9.6 µm), while the size of negative cells was 15 ± 4.22 µm, on average (median 14 µm) (t value -9.76; d = 123; P < 0.001) (Fig. 6B, C).

**Fig. 7:**
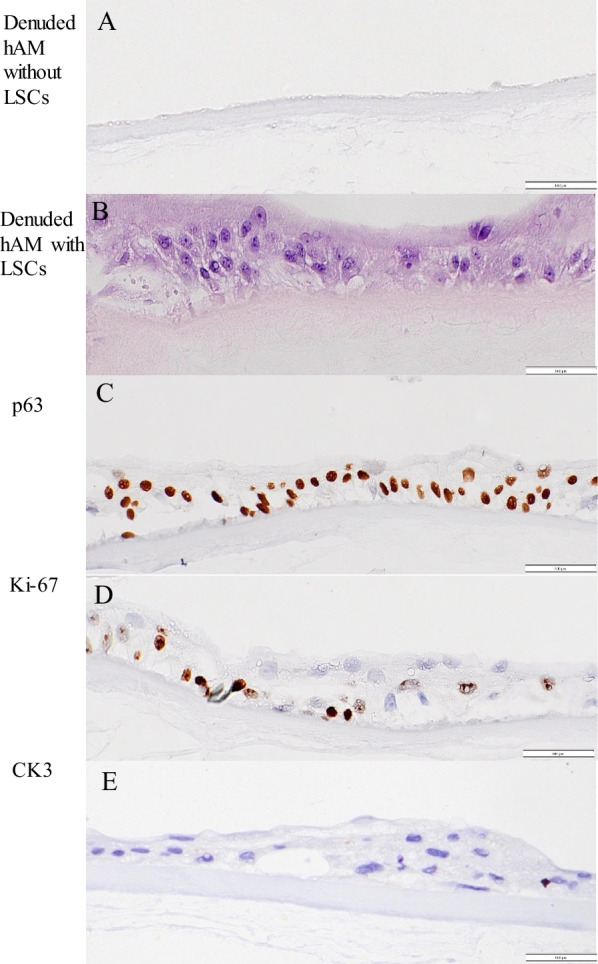
Microphotographs of limbal graft. **A** Hematoxylin–eosin (H&E) staining of denuded human amniotic membrane (hAM) without limbal cells (LSCs); **B** H&E of denuded hAM with LSC that formed multilayer epithelial structure; **C** with immunohistochemically p63-stained positive cells; **D** Ki67-stained positive cells on limbal graft; **E** and CK3-negative stained cells on limbal graft (magnification 100x)

The same primary antibodies were used to demonstrate the presence of p63, Ki67 and CK3 in LSCs grown on hAM. Positive staining for p63 was observed in almost the same percentage in cells grown in culture (71.0 ± 11.3%) as in those grown on the hAM (78.7 ± 9.4%) (Figs. 2A and 7C). Regarding the nuclear positivity for the cell proliferation marker Ki67, our results show the expression of Ki67 in 41.0 ± 12.6% of cells grown in culture and in 40.7 ± 15.9% of cells grown on hAM (t-test 0.233; P = 0.821) (Figs. 2B and 7D). LSCs grown in culture or on hAM did not show any positivity by IHC or ICC for the differentiated cell marker CK3 (Fig. 7E).

### Clinical application of limbal grafts

**Fig. 8:**
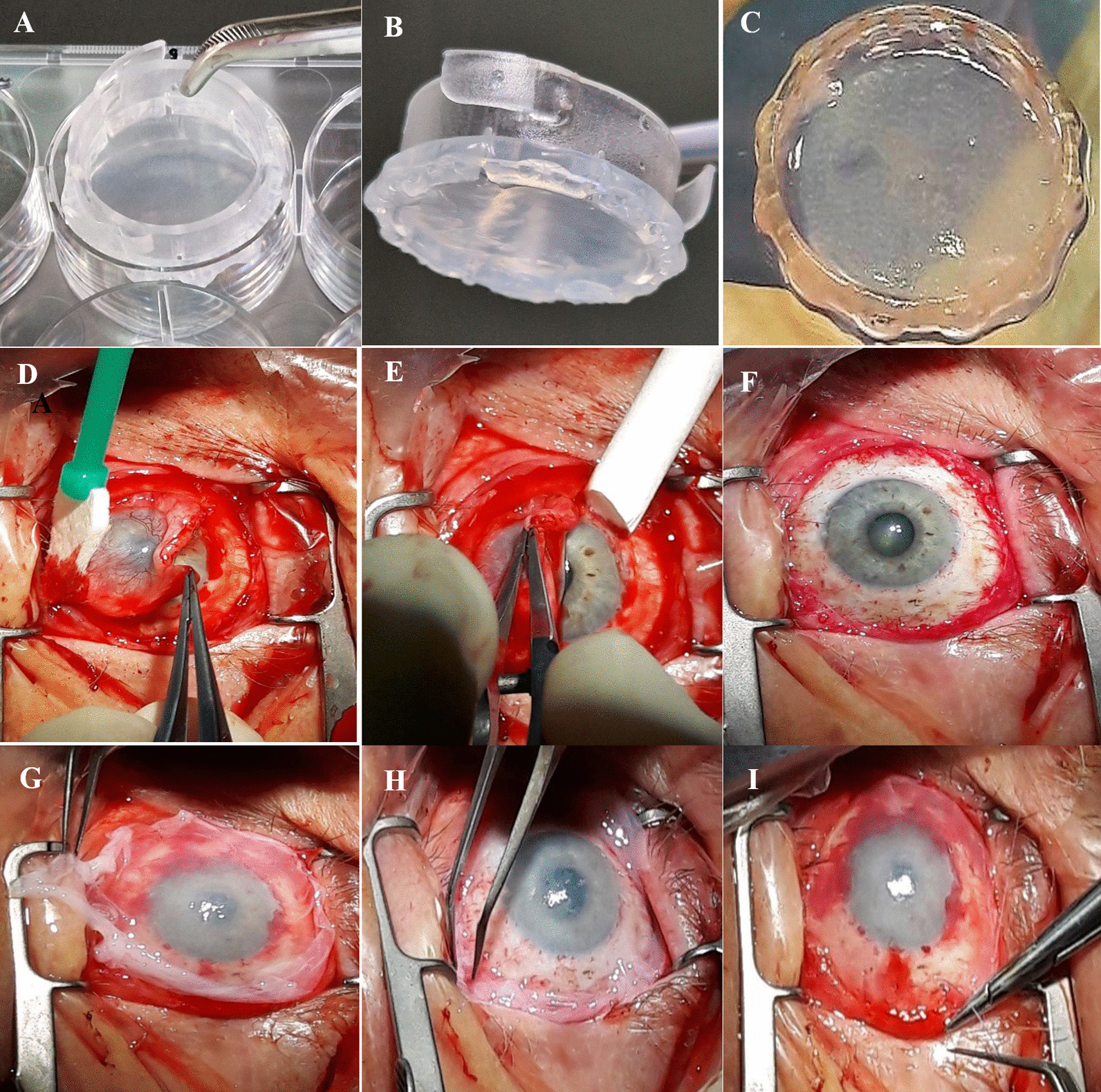
Limbal Graft Transplantation. **A,** **B** The denuded human amniotic membrane (hAM) placed on the 6-well culture dish; **C** HAM, together with limbal stem cells (LSC), separated from one section of the ring (CellCrown6) before clinical application; **D**, **E**, **F** Eye preparation, involving the removal of abnormal fibrovascular tissue; **G**, **H**, **I** Clinical implementation of autologous  (LSC) onto denuded amniotic membrane, prepared as limbal grafts

**Fig. 9:**
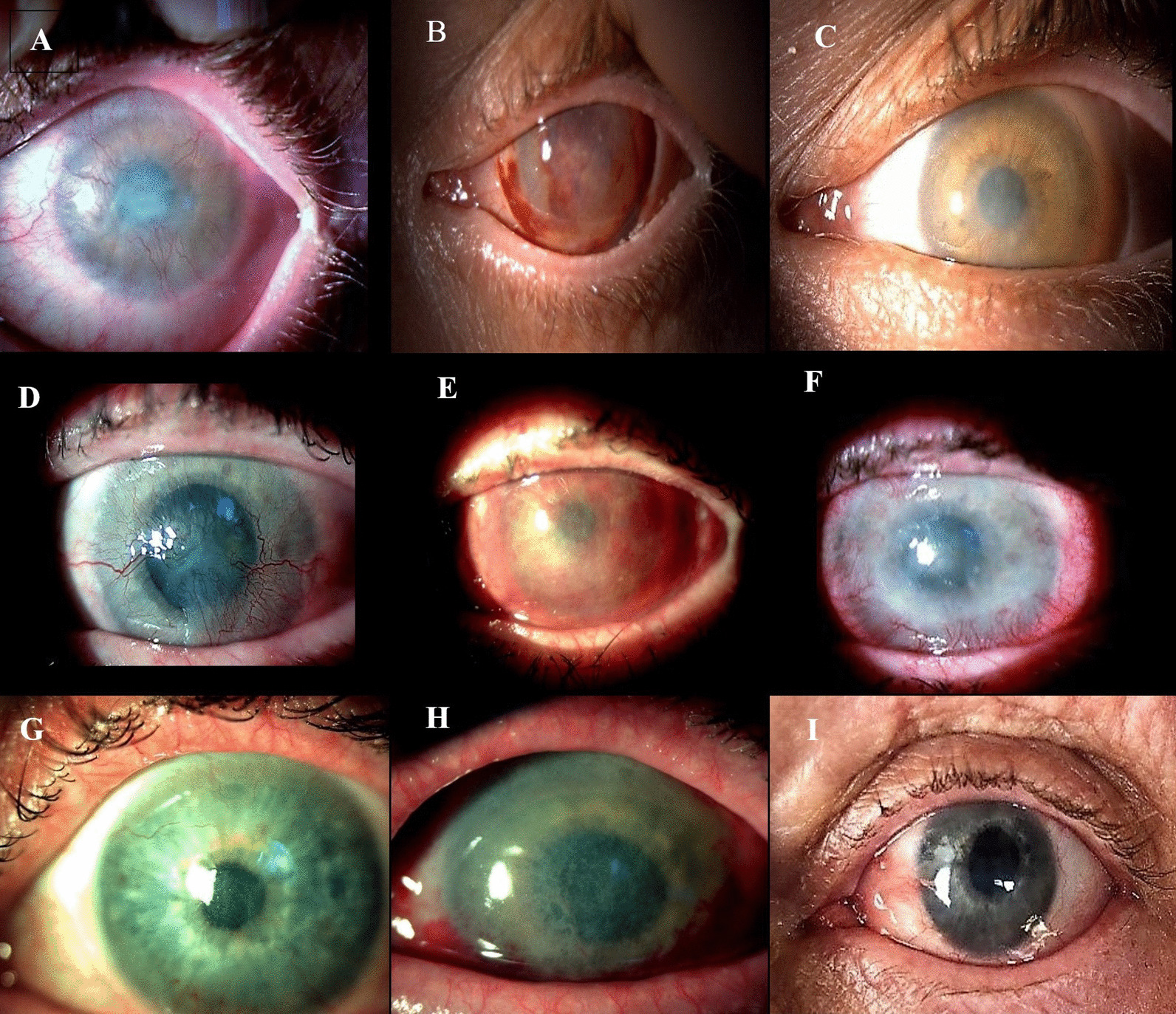
Preoperative and postoperative autologous cultivated limbal epithelial transplantation (CLET) photographs; **A**, **D**, **G **Preoperative slit-lamp photographs of the patient's eye before CLET; (**A**—Total LSCD, **D**—Total LSCD, and **G**—Partial LSCD); **B**, **E**, **H** Photographs taken 45 days postoperatively; **C**, **F**, **I** Photographs taken 12 months postoperatively

Seven patients who had been diagnosed with LSCD underwent CLET treatment for a single eye. All were males with a median age of 43.8 ± 11.9 years (range 28–61). Six patients had unilateral disease, while one had bilateral disease, making a total of seven patients enrolled in this study. Total limbal stem cell deficiency was diagnosed in six patients (Fig. 9A, D), while one had partial limbal stem cell deficiency (covering approximately one-fourth to one-third of the limbus) (Fig. 9G). In all patients, LSCD resulted from an alkaline chemical burn. For each patient, two limbal grafts were transplanted (Fig. 8A–C). Before the clinical application of limbal grafts, it was necessary to remove all abnormal fibrovascular tissue on the affected eye (Fig. 8D–F). The first transplanted graft was placed on the cornea with the limbal cells oriented downward (toward the cornea), while the second had limbal cells facing upward (Fig. 8G–I). Clinical success was defined as a stable corneal surface with the persistence of intact epithelium (with or without peripheral conjunctivalization). Histopathologic success was determined by the absence of goblet cells on the corneal surface, typically found in the conjunctiva, as detected through impression cytology. The mean postoperative follow-up was 25.25 ± 8.8 months. In three patients, eyelid correction and symblepharon surgery were required before CLET. Postoperative treatment included systemic doxycycline 200 mg for two weeks, systemic prednisolone 25 mg for two weeks, preservative-free 0.335% hydrocortisone sodium phosphate 3x/day for two weeks, and tapered over the next two weeks, along with preservative-free artificial tears. Postoperative examinations were conducted on the third postoperative day, 14 and 45 days postoperatively, and at 6 and 12 months (Fig. 9). Clinical success was achieved in five patients with stable epithelialization and significant improvements in vision (Fig. 9C, F, I). One case had reconjunctivalization of the cornea, and one had recurrent epithelial defects. Histopathologic success was achieved in four patients, while three showed goblet cells in the central cornea (Fig. 9F). Two patients are scheduled for corneal transplantation. Best-corrected visual acuity was determined with a Snellen chart. Visual acuity light perception, hand motion, and count fingers were converted to a Snellen equivalent. Visual acuity improved in five eyes after CLET, while it remained unchanged in two eyes. Best-corrected visual acuity (BCVA) increased from 0.037 ± 0.049, at the initial visit to 0.2 ± 0.228 at 12 months after CLET (shown as mean ± SD of all patients) [36].

### Microbiological analysis

An eye swab taken before limbus biopsy and immediately before transplantation showed the current microflora in the patient. There were isolated microorganisms that belong to the normal skin flora, coagulase-negative staphylococci, *Staphylococcus epidermidis*, *Cutibacterium acnes*, and *Staphylococcus aureus,* which is a pathogenic bacterium (Table 3). Microorganisms isolated from these swab samples did not affect the transport medium, and all microbiological results were sterile. Results of analysis of additional samples of 5% antibiotic wash media, GM during cell growth in p0 and p1, hAM swabs after thawing, and GM from limbal grafts immediately prior to clinical use showed that all were sterile.

**Table 3:** Microbiological profile of the eye swab

Patients	Eye swab before the biopsy	Eye swab before clinical application of the grafts
Patient 1	CoNS	CoNS
Patient 2	Sterile	/
Patient 3	CoNS	*Staphylococcus epidermidis*
Patient 4	CoNS; *Bacillus spp.*	*Staphylococcus aureus;* CoNS
Patient 5	*Cutibacterium acnes*	*Cutibacterium acnes*
Patient 6	*Staphylococcus epidermidis*	CoNS
Patient 7	*Staphylococcus aureus*	*Cutibacterium acnes*

### Evaluation of bacterial endotoxin analysis

To evaluate the bacterial endotoxin assay with a valid result, the coefficient of variation (CV) between sample and spike replicates should be ≤ 25% according to the manufacturer; however, the results obtained were < 10%, which is consistent with the results reported by Viganò M, Budelli et al. [37]. Additional acceptable criteria associated with MDV were 1:40, the rate of increase in recovery was between 50 and 200%, and the onset time of the NTC and no spiked sample was longer than the onset time λ. An attempt was made to find a suitable dilution that does not activate and/or inhibit the enzymatic reaction. The endotoxin limit (EL) results presented were < 0.200 EU/device according to FDA guidelines for solid anterior segment devices (USP < 1085 >) [38].The best endotoxin results in limbal grafts (< 0.125 EU/mL) were achieved using a higher sensitivity cartridge (0.5–0.005 EU/mL) and 1:25 dilution (Table 4).

**Table 4:** Endotoxins measured in limbal graft (n = 6) with sensitivity cartridge (0.5–0.005 EU/mL)

Sample ID	20LSC2 in duplicate	22LSC1 in duplicate	22LSC2 in duplicate	Acceptance criteria
Dilution	25	25	25	40
CV between sample replicates 1/3 (%)	0	0	0	25
CV between spike replicates 2/4 (%)	6.85	4.3	8	25
Spike Recovery 2/4 (%)	108	187	138.5	50—200
Sample value (EU/mL)	< 0.125	< 0.125	< 0.125	< 0.200

## Discussion

Tissue engineering technology has been gaining importance during the last three decades as a major part of regenerative medicine that moves stem cell biology from laboratory research to clinical practice. Limbal graft production before clinical application has limited time to perform all essential analyses to check the necessary markers for identity, potency, stemness, proliferation rate and tumorigenicity. Despite the fact that we validated the method and obtained the license of the Ministry of Health for ATMP as a “hospital exemption”, failure can occur in such an innovative therapy, so it was necessary to demonstrate consistency in process with established quality indicators and periodically analyze them to the smallest detail.

In clinical application, there are different methods of isolation of LSCs (dissociation with trypsin/EDTA or dispase digestion), with or without fibroblasts as a feeder layer, using hAM or fibrin as a carrier of limbal cells [39, 40]. Currently, there is no consensus or harmonization among different laboratories about methods with ATMP because each method has advantages and disadvantages. In our study, a method was selected that was described by Schwab IR in 2000 [41]. Cultivation of autologous LSC started after a small limbal biopsy was taken from the ocular surface of a healthy contralateral eye and expanded in vitro on 3T3 feeder layer cells on denuded hAM. After they successfully adhere to the hAM, LSCs must adapt and proliferate without losing their stemness or proliferative potential and must create progressively growing, strongly adhesive colonies that push away the surrounding feeder layer cells.

Sample collection, processing time, and the method of enzymatic digestion of the limbus with the yield, viability, PD, cPDs, CFE, p63 and Ki67 positive cells were described as part of potency. They are important as initial quality indicators of the success of growing a sufficient number of LSC used as starting materials for the manufacture of ATMPs. When processing human tissue such as the biopsy material of the limbus, the initial number of cells is very low, and repeating the biopsy is not an option. If a limbus biopsy of approximately 2 mm^2^ is taken, the expected number of cells to be obtained after triple enzymatic digestion should be known, and if the expected number is not obtained, the reasons for inconsistency must be critically evaluated. These results indicate that consistency and optimal sample quality are crucial to obtain a sufficient amount of LSCs for the preparation of two limbal grafts for transplantation, one graft for quality control tests and the rest of the cells for freezing in liquid nitrogen in case of the need for recultivation. The CFE method can be used to determine the possibility of proliferation, the ability to differentiate, as well as the efficiency of colony formation. It is crucial that there is a sufficient number of stem cells (holoclones) in the cell culture in order to ensure long-term regeneration of the epithelium. Proliferative capacity decreases from holoclones (stem cells) to meroclones (progenitors) and paraclones transient amplifying (TA) that have the least stem cell potential. During in vitro limbal cell culturing, holoclones progressively lose their proliferative capacity and become differentiated, TA with limited division potential (paraclones).

For LSC as adult stem cells, basic morphological parameters to establish the key indicator of quality control may be a useful tool in combination with other laboratory analyses. The results of our measurements of basic morphological parameters of LSC during the cultured state suggest that the nuclear size scales strongly with the cell size across a population in culture, as expected from the previous data on multiple types of human cells [32]. The observed inverse correlation of p63 levels with nuclear and cell size, which is associated with the stemness of LSC [42], indicates that only a subfraction of cells within holoclones are stem cells with huge self-renewal potential. Indeed, it was previously demonstrated that the density of p63 protein within LSCs, rather than its mere presence, is directly correlated with the LSC population that comprises 5–7% of the total population of LSC in culture [43, 44]. Our results of cell size measurements of LSC in their culture environment are in agreement with earlier reports [45]. As previous reports found that the cell area fluctuates during the course of LSC passaging due to cell jamming mechanisms in cultured colonies, the stem cells analyzed in our study were most likely out of the jamming phase because their sizes were similar to previously measured sizes of cells that were out of the jamming phase, possibly reflecting high densities of LSC in the cultures analyzed in our study [45].

Measurements of mitotic spindle morphology and dynamics are scarce in the literature on stem cells. To our knowledge, this study presents the first measurements of basic parameters of mitotic spindles in LSCs grown in vitro in their near-native conditions, and it is one of the first representations of stem cells imaged by live-cell microscopy with tubulin and DNA dyes. The mitotic spindles observed in LSCs were somewhat smaller than the metaphase spindles of differentiated human cells, even when compared to epithelial cells isolated from the human retina, that is, the retinal pigment epithelium-1 (RPE-1) cell line [46]. However, the spindle lengths in LSCs were not drastically smaller than those in the conventional human cell lines used in culture. This is unexpected, as the data presented for human neural stem cells [47] and human embryonic stem cells (hESCs) [48] reported spindles in stem cells ∼7 μm in length and ∼12 μm in width. Interestingly, almost exactly the opposite relation of length and width in human LSCs in culture was observed in our study, both in fixed and live-imaged cells, giving rise to rather elongated spindles similar to those observed in RPE-1 cells [33]. An explanation for these discrepancies could be related to the usage of methanol fixations in the studies on stem cells. Methanol fixation at low temperatures (− 20 °C) can reduce the length of the mitotic spindle and increase its width, thus resulting in characteristically shaped spindles that have a larger width than length, similar to cold treatments in general. Such phenomena are not observed when cells are fixed in PFA and glucopyranosyl lipid adjuvant (GLA) solution at 37 °C [49], a protocol used in the present study (see Methods). Alternatively, the differences could be a result of variability within stem cells from different tissues or variances in differentiation levels between different stem cell models used in different studies. In this study, we provided parameters for the mitotic process in LSCs cultures whose duplicates were used to prepare limbal grafts that were used for CLET. As the clinical success of transplantation was high, we argue that the mitotic parameters measured in the duplicates provide a good estimate of the ranges that are relevant for successful clinical practice during CLET. In this sense, the extent of genetic instability in the LSC population, which was less than 5% in multiple parameters, could be used as a reference point for future studies in LSCs or other types of stem cells used in cell therapy approaches, presuming the use of similar culturing methods. Similarly, our observation that the spindle length in LSCs ranged from 8 to 15 μm, and the spindle width from 6 to 12 μm could be used as an indication of the quality range of these parameters. It would be valuable to establish in future studies the absolute ranges of the relevant mitotic parameters for clinical applications, which would require a large sample size, both in the number of donors and cells and in the different genetic backgrounds of the donors.

Human diploid nontransformed but immortalized cell lines such as RPE-1 are recognized for their high karyotype stability and low level of defective mitotic phenomena, including lagging chromosomes, acentric chromatin fragments and chromatin bridges [50]. Interestingly, the level of defective mitosis observed in our study during anaphase in LSCs grown in culture (< 4%) is comparable to that in RPE-1 cells grown in culture and scored during anaphase (< 5–6%) [50, 51]. Similar percentages in LSCs and RPE-1 cells were observed for transiently multipolar mitotes, measured during metaphase (< 3% in both cases) [52]. Transient multipolarity can occur by multiple mechanisms, chiefly overduplication of centrioles, cytokinesis failure [53], and loss of spindle pole integrity [54]. Interestingly, the low number of defective mitotic phenomena observed here is contrary to a recent report that human pluripotent stem cells (hPSCs) frequently become aneuploid with abnormal chromosome numbers due to a high number of mitotic chromosome segregation errors during propagation of hPSCs in culture [51]. This could be related to the surprising point raised by the same study that the development potential negatively influences mitotic fidelity, at least in hPSCs [51]. One could argue that the differences observed between hPSCs and noninduced human stem cells from adult tissues, such as the LSCs studied here, reflect differences in the induced and noninduced stemness of cells.

The biosafety of LSCs was also explored in our study from many aspects. The percentage of interphase errors observed and quantified in LSCs in our study was low (< 2.5%) and comparable to the frequency of the micronuclei observed in RPE-1 cells grown in culture and scored during interphase (< 2–3%) [55]. Furthermore, the frequency of interphase errors is similar when compared to RPE-1 s with abnormal nuclear shapes (< 5%) [56] and binucleated interphase RPE-1 cells in their native tissue environment (< 3%) [57]. Regarding propagation of rare LSCs in which we observed abnormal mitosis or interphase, these phenomena were reported to typically induce strong cell cycle arrest, apoptosis, or senescence in non-transformed cells [34], effectively limiting the propagation of abnormal cells [19, 34, 58]. Thus, based on these data and the high success rate of the grafts whose duplicates were analyzed in this study, we speculate that LSCs with abnormal karyotypes that are the result of aberrant mitoses would be selected against after transplantation of LSCs into the organism, thus not affecting the success of LSC therapy. Taken together, the data presented here demonstrate that when grown in culture by the protocols presented in this study, LSCs are not prone to chromosome mis-segregation or cytokinesis failure and thus to aneuploidy and polyploidy. While currently used methods such as karyotyping and SNP genotyping still represent the golden standards for genetic stability testing in clinic we reason that the presented methodology, based on analysis of chromosomal aberrations during mitosis, could be used as a complementary approach with different benefits and limitations. Thus, while karyotyping certainly offers more in-depth analysis of chromosomal changes, the methodology is still too laborious and expensive for daily use in a clinical setting and uses a relatively low number of cells. On the other hand, analysis of chromosomal instability in the context of mitosis, which is by far the most dominant mechanism of induction of aneuploidy, especially in the context of cancer, is a relatively simple and inexpensive method that could be used to quickly estimate the extent of chromosomal instability in a large number of cells and thus their pro-tumorigenic potential [59].

The epithelial stem cell marker p63 (clones ΔNp63α and 4A4) was used to determine the percentage of stem cells in all stages of cultivation. Precise determination of the percentage of stem cells in cultured limbal grafts is considered together with reliable quality control to ensure optimal therapy. Expression of the stemness marker p63 can be analyzed in single-cell suspension, cell culture and limbal grafts. By comparing the methods of FCM and fluorescence microscopy, we obtained a similarly high percentage of p63-positive cells (Figs. 3 and 5C) and slightly lower for the ICC/IHC methods (Figs. 2A, 7C). No statistically significant difference in the percentage of positive cells was determined by IHC or ICC methods for Ki67 in cultured cells (Fig. 2B) or in limbal grafts (Fig. 7D).

There is no single specific marker for LSC. Markers such as the presence of ΔNp63α and ABCG2 and the absence of corneal epithelial differentiation markers such as CK3 can be used to identify LSCs [60]. Under resting conditions, basal limbal epithelial cells, which represent the epithelial stem cell pull, exclusively express ΔNp63α [4]. Interestingly, in isolated/expanded limbal cell cultures and limbal tissue explantants grown in vitro, ΔNp63α could be detected in a majority of cells, including basal and suprabasal limbal cells, challenging ΔNp63α as the “one and only” limbal stem marker. All holoclone-forming limbal cells are positive for ΔNp63α but also a substantial percentage of meroclonal cells. Therefore, additional parameters, such as the intensity of ΔNp63α nuclear expression or the clonogenisity of LSCs, are useful indicators to distinguish LSC subclones in vitro. Our FCM analysis showed a high percentage of ΔNp63α-expressing cells expanded in vitro (Fig. 5C). This corroborates the findings of Di Iorio et al. [4].

Although FCM has great potential in the characterization of ΔNp63α expression in limbal cells, it cannot measure the basic morphological parameters because the staining protocol includes the permeabilization of cells in a solution that irreversibly decreases cell morphology (size and granularity). Contrary to IHC, high and low ΔNp63α expression subpopulations could not be distinguished by using a ΔNp63α-specific antibody (clone C-12). Over the years, additional stem cell-associated markers such as ABCG2 and ATP binding cassette subfamily G member 5 (ABCG5) have been studied with the intent of characterizing LSCs. In contrast to ΔNp63α, ABCG2 is expressed only on basal limbal cells but is omitted from the central and peripheral cornea, thus emphasizing the role of ABCG2 as a marker of small resting LSCs. Interestingly, our FCM analysis confirmed that ABCG2-positive LSCs although rare in a population of expanded cultures are small cells based on the FSC value (Fig. 5D) and could represent true long-lived LSCs.

The results above as part of the impurity in the ATMP product can also be assessed with molecular biology techniques. With ddPCR, the absolute quantification of 3T3 DNA is possible in human DNA originating from LSCs. Therefore, ddPCR is one of the most sensitive techniques for absolute quantification and is used for the quantification of DNA that is present in small amounts, such as cell-free DNA, in liquid biopsy of cancerous patients [26]. The ddPCR method is extremely useful when a search is performed for target DNA in an abundant DNA background. This principle was already used in GMO detection [61] or in the clinical setting of detecting minimal residual disease of various cancers [62]. It is also extremely useful when complex matrices are present in the sample, so it was also introduced as a method in residual DNA testing in the process of manufacturing biological drugs [63]. However, ddPCR enables high-throughput analysis and requires a small sample size compared to FCM.

The methods used to analyze cells for clinical use are ICC/IHC because they combine immunological, histological and biochemical methods in the identification of specific tissue components based on specific antibody binding. With these methods, different cell types can be identified simultaneously, e.g., stem cells with p63 stem cell marker expression and Ki67 in mitotically active cells or differentiated epithelial cells expressing CK3. In vitro, measurements of LSC established in limbal grafts were evaluated. Our results show that LSCs in limbal grafts positive for p63 had a small size with a median of 9.6 µm, while negative cells had a median of 14 µm.

According to Good Manufacturing Practice (GMP), LSCs grown in laboratory conditions should be produced in clean room laboratories and require a class A MSC for each manipulation step. These GMP practices provide that the ATMP can be produced in safe (microbiology sterile, control of bacterial endotoxin and mycoplasma contaminations) and good quality control conditions. The microbiological quality and safety of LSCs is very important because microbiological contamination of transplanted cells can consequently cause eye infection and even more severe eye damage. It is extremely important to carry out microbiological control of every procedure in obtaining a quality transplant, all for the purpose of effective treatment of patients with eye damage. Appropriate antimicrobial therapy with drops should also be applied to the eye where we transplanted the limbal graft.

Endotoxins are a group of pyrogens, i.e., substances produced by gram-negative bacteria that cause a rise in body temperature in humans [64] and many other complications, such as inflammatory processes in the eye. After cell death, gram-negative bacteria release endotoxins into host cells. LPS can have toxic effects on humans and animals [65] and play a key role in bacterial-host interactions by influencing host immune responses. Humans respond to high endotoxin contractions by producing antibodies. Due to the dangerous effects that high concentrations of endotoxins can have on human health, it is necessary to follow the requirements of the European Pharmacopeia Chapter 2.6.14., i.e., applied the Bacterial Endotoxin Test (BET) [29]. Because of their high thermal stability, endotoxins are difficult to destroy by sterilization and can withstand large differences in pH. It is very important to validate the endotoxin detection method before clinical application and to define what is an acceptable dilution for LAL analysis. According to European Medicines Agency (EMA) regulations [66], limbal grafts are safe for clinical use if the endotoxin level is < 0.500 EU/mL. However, a dilution of 1:25 resulted in an even higher and more accurate bacterial endotoxin value of < 0.125 EU/mL is achieved, which is in line with the FDA guidelines (< 0.200 EU/mL) of the Recommendation for Endotoxin Testing for single use of an intraocular ophthalmic device [38].

Transplantation of the hAM in the treatment of the cornea with the aim of restoring the damaged limbal stroma was first described by Kim and Tseng in 1995 [67]. Since then, the clinical use of hAM has grown worldwide. Further research has shown that the use of hAM leads to faster healing and reduced pain, inflammation and stromal scarring [68]. For the above reason, denuded hAM has been used as a carrier for LSC, and its preparation as a limbal graft helps in the expansion of LSC in vivo. Other clinical centers have also recognized the utility of hAM in the treatment of patients with LSCD [41, 69–71]. Scientists have proven in studies that intact hAM can preserve the phenotype of the limbal epithelium in vitro, unlike denuded hAM [72]. Some experts suspect that feeder layers function more like surrogate niche cells [68]; that is, they can preserve the status of LSC with regard to the maintenance of slow cycle properties and positive expression of p63 [73]. These results indicate the necessity of using 3T3 or human mesenchymal stem cells as a feeder layer when denuded hAM is used as a limbal cell carrier [68, 74].

Human stem cells play an important role in regenerative medicine, and the presence of feeder layer cells is required for stem cells to grow and differentiate. Cultures of adherent growth-arrested 3T3 murine embryonic fibroblasts have been used as feeder layer for more than 60 years to promote stem cell proliferation [75]. The most commonly used methods for growth arrest feeder cells remain mitomycin-C or γ-irradiation treatments [76]. Both treatments cause irreversible cell cycle arrest and might be necessary for epithelial cell support [77]. Experimental evidence indicates that these metabolically active cells are unable to divide but are still viable and bioactive and provide growth factors to the culture media to help stem cells proliferate. This is not the only way that feeder cells promote the growth of target cells; there are several epithelial cells, and they need physical contact with a feeder layer for expansion and survival [78]. Feeder layer cells also have a significant impact on other processes, such as synthetizing extracellular matrix proteins necessary for cell growth and acting as a substrate for the attachment of adherent cells [79]. In addition, some studies suggest that soluble factors produced by 3T3 fibroblasts prepared as a feeder layer could be involved in the promotion of niche regulation of LSCs [68]. Our results agreed with prior publications discussing 3T3 cells prepared as a feeder layer in limbal grafts, as impurities were low (1.79% ± 0.241). Long-term follow-up of clinical experience (over five years of follow-up) and posttreatment analysis showed that cultured epithelial autografts with 3T3 coculture are safe and have benefited thousands of patients worldwide [80, 81].

In this study, CLET was performed on seven eyes where LSCD resulted from an alkaline burn, with six out of seven eyes exhibiting total LSCD. Clinical success was achieved in five patients, while histopathological success was achieved in four patients. Clinical success was not achieved in two patients who had an exceptionally severe alkaline injury with symblepharon involving both eyelids, and a repeat procedure is planned. Prabhasawat and colleagues reported clinical and pathological success rates of 77.9% and 72.3%, respectively [82]. Similar results were demonstrated in this study and are in line with results reported by other authors, with success rates ranging from 57 to 78% [83–85]. One limitation of this study is the small number of patients, underscoring the need for further research with a larger cohort of patients who have experienced the same chemical injury. The objective of CLET is to improve the corneal surface, reduce conjunctival inflammation, eliminate symblepharon, and provide better conditions for subsequent keratoplasty, thereby increasing the chances of corneal graft success. Patients with total LSCD who had the highest cell yield after triple enzymatic digestion, exceeding > 100 000 cells (SD for all samples 93 203 ± 37), were the youngest patients (28 and 30 years old). We emphasize that these patients experienced significant visual improvement (Fig. 9A–F) and had cell viability exceeding > 90%. The effectiveness of the therapy was impacted by the patient's initial condition, and the most favorable clinical results were observed in patients with partial LSCD (Fig. 9G–I).

An additional limitation of this study arises from the fact that a method was not developed that would use a culture medium completely free of animal products.

## Conclusion

In this study, the expansion of LSCs used in medical treatments was described, which showed stable stemness in all stages of cultivation, i.e., in a single-cell suspension, cell culture and limbal grafts. Several different laboratory methods show a high prevalence of p63-positive LSCs that undergo the mitotic process with extremely high fidelity, which indicates a high stability of the karyotype, which is in favor of quality and safe eye regeneration therapy. Data on the mitotic spindles of stem cells are rarely described in the scientific literature, especially in relation to LSCs. Therefore, in this manuscript, we quantified the basic parameters of the mitotic process and the morphology of the mitotic spindle of LSCs grown in culture during the preparation of limbal grafts which are used in direct clinical applications.

## Data Availability

The data underlying this article will be shared on reasonable request to the corresponding author.

## Abbreviations

2D: Two-dimensional
3D: Three-dimensional
4D: Four-dimensional
ABCG2: ATP-binding cassette subfamily G member 2
ABCG5: ATP-binding cassette subfamily G member 5
APC: Allophycocyanin
ATMPs: Advanced therapy medicinal products
BCVA: Best-corrected visual acuity
BET: Bacterial endotoxins test
CFE: Colony-forming efficiency
CK3: Cytokeratin 3
CLET: Cultivated limbal epithelial transplantation
cPDs: Cumulative population doubling
CT: Total number of culture days
CV: Coefficient of variation
DAB: 3,3′-Diaminobenzidine
DAPI: 4′,6-Diamidino-2-phenylindole
ddPCR: Droplet digital polymerase chain reaction
DMEM: Dulbecco's modified eagle medium
DMSO: Dimethyl sulfoxide
DNA: Deoxyribonucleic acid
DPBS: Dulbecco’s phosphate buffer saline calcium and magnesium free
DT: Doubling time
EDQM: European Directorate for the Quality of Medicines & HealthCare of the Council of Europe
EDTA: Ethylenediaminetetraacetic acid
EGF: Epidermal growth factor
EL: Endotoxin limit
EMA: European Medicines Agency
EU: Endotoxin units
FBS: Fetal bovine serum
FCM: Flow cytometry
FDA: Food and Drug Administration
FSC: Forward scatter
GA: Glutaraldehyde
GLA: Glucopyranosyl lipid adjuvant
GM: Growth medium
GMP: Good manufacturing practice
H&E: Hematoxylin and eosin
hAM: Human amniotic membrane
hESCs: Human embryonic stem cells
ICC: Immunocytochemical
IHC: Immunohistochemical
LAL: Lysate of amebocyte limulus test
LPS: Lipopolysaccharide
LSC: Limbal stem cells
LSCD: Limbal stem cell deficiency
MDV: Maximum dilution valid
MSC: Microbiological safety cabinet
NGS: Normal goat serum
NTC: Negative control
p63: Tumor protein 63
PE: Peridinyl chlorophyllin
PFA: Paraformaldehyde
RPE-1: Retinal pigment epithelium-1
sCMOS: Scientific complementary metal oxide semiconductor
SD: Standard deviation
SSC: Side scatter
TA: Transient amplifying
TRIS: Tris(hydroxymethyl)aminomethane
